# NatB Domain-Containing CRA-1 Antagonizes Hydrolase ACER-1 Linking Acetyl-CoA Metabolism to the Initiation of Recombination during *C*. *elegans* Meiosis

**DOI:** 10.1371/journal.pgen.1005029

**Published:** 2015-03-13

**Authors:** Jinmin Gao, Hyun-Min Kim, Andrew E. Elia, Stephen J. Elledge, Monica P. Colaiácovo

**Affiliations:** Department of Genetics, Harvard Medical School, Boston, Massachusetts, United States of America; University of Vienna, AUSTRIA

## Abstract

The formation of DNA double-strand breaks (DSBs) must take place during meiosis to ensure the formation of crossovers, which are required for accurate chromosome segregation, therefore avoiding aneuploidy. However, DSB formation must be tightly regulated to maintain genomic integrity. How this regulation operates in the context of different chromatin architectures and accessibility, and how it is linked to metabolic pathways, is not understood. We show here that global histone acetylation levels undergo changes throughout meiotic progression. Moreover, perturbations to global histone acetylation levels are accompanied by changes in the frequency of DSB formation in *C*. *elegans*. We provide evidence that the regulation of histone acetylation requires CRA-1, a NatB domain-containing protein homologous to human NAA25, which controls the levels of acetyl-Coenzyme A (acetyl-CoA) by antagonizing ACER-1, a previously unknown and conserved acetyl-CoA hydrolase. CRA-1 is in turn negatively regulated by XND-1, an AT-hook containing protein. We propose that this newly defined protein network links acetyl-CoA metabolism to meiotic DSB formation via modulation of global histone acetylation.

## Author Summary

Achieving accurate chromosome segregation is a critical outcome for any cell division process. Programmed DNA double-strand break formation is a central mechanism set in place to promote faithful chromosome segregation during meiosis. A subset of these DSBs is repaired as crossovers via reciprocal exchange of genetic information between homologous chromosomes resulting in physical attachments (chiasmata) between homologs, which ensure proper chromosome alignment at the metaphase plate at meiosis I, and also promote genetic diversity. How this regulation operates in the context of different chromatin architectures and accessibility, and how it is linked to metabolic pathways, is not understood. In this study, we found that CRA-1, a NatB domain-containing protein, promotes histone acetylation by maintaining the levels of acetyl-Coenzyme A (acetyl-CoA) through antagonizing ACER-1, a previously unknown and conserved acetyl-CoA hydrolase. CRA-1 is in turn negatively regulated by XND-1, an AT-hook containing protein. We leveraged this discovery to find a connection between the levels of acetyl-CoA, histone acetylation and DSB formation. We identified a novel protein network that links the regulation of DSB formation to the modulation of global levels of histone acetylation, and revealed a link between metabolism and the regulation of DSB formation.

## Introduction

Achieving accurate chromosome segregation is a critical outcome for any cell division process. This is strikingly evident during the meiotic cell divisions leading to the formation of haploid gametes (i.e. oocytes and sperm), where errors in chromosome segregation result in aneuploidy and therefore increased miscarriages, infertility, birth defects and tumorigenesis in humans [1,2]. A central mechanism set in place to promote faithful segregation during meiosis consists in the formation of programmed meiotic DNA double-strand breaks (DSBs) via a conserved topoisomerase-like protein, Spo11, and its associated factors [3–8]. Meiotic chromosomes are organized into arrays of loops attached to chromosome axes, with studies in yeast and mice indicating that programmed DSBs form in loop sequences that are tethered to the axes [9–12]. A subset of these DSBs is repaired as crossovers via reciprocal exchange of genetic information between homologous chromosomes resulting in physical attachments (chiasmata) between homologs. Ensuring crossover formation therefore serves two critical purposes: to ensure genetic diversity and to hold homologs together so they can properly align at the metaphase plate and then accurately segregate away from each other at meiosis I. However, despite the critical importance of DSB formation to human reproductive health, the mechanisms regulating meiotic DSB frequency and distribution throughout the genome remain poorly understood.

One plausible mode of DSB regulation is via alterations in chromatin accessibility. In that vein, post-translational modifications such as histone methylation have been recently implicated in this process by the analysis of DSB hotspots (discrete regions with high frequencies of recombination initiation) [13]. Studies in yeast have suggested that an open chromatin structure is required for Spo11 to access its DNA substrate to generate DSBs [14]. Recent mapping of meiotic recombination initiation sites in yeast and mice has suggested trimethylated lysine 4 on histone H3 (H3K4me3) as a critical modification required for meiotic DSB formation [15,16]. However, H3K4me3 enrichment and DSB formation showed little correlation in these organisms [17–19]. Therefore, the chromatin state requirements promoting DSB formation remain a key unresolved question.

Importantly, not much is known about the roles of histone acetylation in modulating DSB frequencies and/or distribution. Acetylated histone H3K9 has been found to be a major hotspot-associated modification in fission yeast, but it has only a mild, albeit significant, effect on DSB formation at hotspots [20]. This raises the possibility that changes to either a combination of histone acetylation marks or to global histone acetylation, instead of just to a specific acetylation mark, might be critical for DSB formation. Histone acetylation is generally associated with euchromatin, it promotes transcriptional activation, and therefore could be a critical level of control for genomic functions. Histone acetylation involves histone acetyltransferases (HATs) and histone deacetylases (HDACs), which either respectively catalyze or reverse the transfer of an acetyl group from acetyl-Coenzyme A (acetyl-CoA) to lysine residues on histones. Recent studies have shown that regulation of acetyl-CoA levels by metabolic enzymes can affect global histone acetylation. In yeast, reducing acetyl-CoA carboxylase expression results in a global increase of histone acetylation [21], and mutation of the acetyl-CoA synthetase Acs2 causes global histone deacetylation [22], suggesting that the regulation of acetyl-CoA levels is another critical control of histone acetylation. However, little is known about how regulation of global histone acetylation is involved in specific cell activities or whether it may modulate biological pathways.

The genetic tractability of the nematode *C*. *elegans* is coupled to various other features that make it an extremely advantageous model system for investigating the regulation of meiotic DSB formation. Nuclei are positioned in a temporal/spatial gradient within the germline, affording ease of simultaneous cytological analysis of all stages of meiotic prophase [23]. High-resolution microscopy utilizing antibodies to well-established markers of DSB repair, coupled with the use of mutants in which DSBs are formed but fail to repair, thereby trapping all DSBs, allows for analysis of DSB frequency and temporal distribution throughout meiotic progression [24–28]. Moreover, the recent identification of two new factors, XND-1 and CRA-1, potentially modulating chromatin architecture and exerting differential control on either DSB or crossover formation between the transcriptionally silenced and highly heterochromatic X chromosome and the transcriptionally active and more euchromatic autosomes, offer a unique opportunity to identify the mechanisms linking changes in chromatin state to the regulation of the frequency and timing of DSB formation. XND-1 is an autosomally-enriched AT-hook domain containing protein proposed to be involved in regulating acetylation of lysine 5 on histone H2A (H2AK5ac) and the global distribution of crossovers, but it affects DSB formation only on the X chromosome [29]. CRA-1 is a NatB domain-containing protein conserved in yeast, worms, flies, zebrafish and mammals, previously shown to promote chromosome synapsis and crossover formation preferentially on the autosomes during meiosis in *C*. *elegans* [30]. NAA25, the CRA-1 homolog in humans, has been suggested to be the non-catalytic subunit of the NatB N-terminal acetyltransferase complex [31]. However, the mechanisms of function for both XND-1 and CRA-1 remained to be determined.

Here we identified changes in global histone acetylation levels throughout the *C*. *elegans* germline and provide evidence that this is mediated by CRA-1, which maintains the levels of acetyl-CoA by associating with and antagonizing the activity of a previously uncharacterized and conserved acetyl-Coenzyme A hydrolase, ACER-1. CRA-1 is autosomally enriched and exhibits an XND-1-dependent, tightly regulated pattern of expression within the germline, which contributes to the dynamic regulation of histone acetylation during meiotic prophase. The physiological significance of this tight regulation of histone acetylation in the germline is underscored by our findings that increased acetyl-CoA and histone acetylation levels are accompanied by increased DSB formation. High-resolution microscopy reveals that sites of early meiotic recombination events are located near chromosome axes, suggesting that DSB formation/repair may involve a tethered loop-axis mechanism in *C*. *elegans*. Taken together, XND-1, CRA-1 and ACER-1 link metabolic functions, accurate chromosome segregation and genomic diversity via the regulation of acetyl-CoA levels, identifying a role for the regulation of global histone acetylation in modulating specific cell biological functions.

## Results

### CRA-1 is required for normal levels of global histone acetylation in the germline

**Fig 1:**
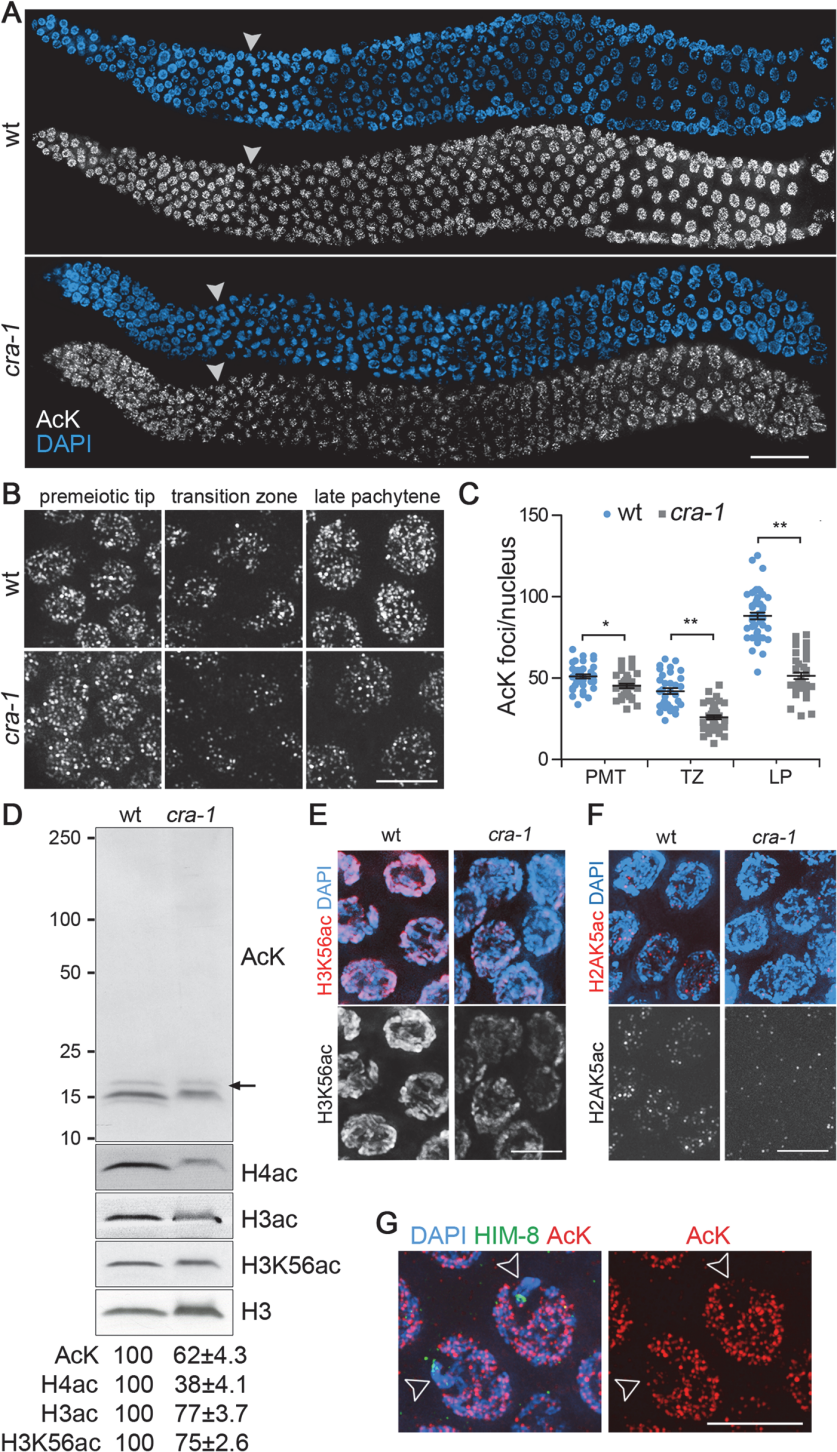
Global histone acetylation is impaired in *cra-1* mutants. (A) Wild type and *cra-1* mutant germlines co-stained with a rabbit pan acetylation antibody (AcK) (white) and DAPI (blue). White arrowheads indicate entrance into meiosis (beginning of the transition zone). Bar, 30 μm. (B) High-magnification images of wild type and *cra-1* mutant germline nuclei immunostained with AcK. Images represent 3D data stacks of whole nuclei. Bar, 5 μm. (C) Quantification of the number of acetylation foci observed per nucleus in (B). PMT, premeiotic tip; TZ, transition zone (leptotene/zygotene); LP, late pachytene. Bars represent the mean number of foci ± SEM. * P = 0.0029, ** P<0.0001, two-tailed Mann-Whitney test, 95% C.I. (D) Western blot analysis of protein lysine acetylation in wild type and *cra-1* mutant whole worm lysates using the AcK, histone H3ac (pan-acetyl), histone H4ac (pan-acetyl) and H3K56ac antibodies. Arrow indicates histone H3 bands. Levels of protein acetylation are measured with ImageJ (National Institutes of Health, USA). Numbers represent mean ± SEM for data from at least four independent experiments. (E) Pachytene nuclei of wild type and *cra-1* mutant gonads were co-stained with anti-H3K56ac antibody (red) and DAPI (blue). Bar, 5 μm. (F) Pachytene nuclei of wild type and *cra-1* mutant gonads were co-stained with anti-H2AK5ac antibody (red) and DAPI (blue). Bar, 5 μm. (G) Pachytene nuclei of wild type gonads co-stained with AcK (red), anti-HIM-8 antibody (X chromosome marker, green) and DAPI (blue). Open arrowheads indicate X chromosomes. Bar, 5 μm.

The presence of a NatB domain in CRA-1 prompted us to examine whether CRA-1 might regulate protein acetylation in the germline. Immunostaining of dissected gonads and Western blot analysis of whole worm lysates with a pan acetylation antibody revealed a decrease in protein lysine acetylation in *cra-1* mutant germlines compared to wild type (Fig. 1A-D). Given that histones comprise a large portion of the proteins that undergo acetylation in the cells, we proceeded to examine whether this decrease might also reflect changes in histone acetylation. Western blot analysis of whole worm lysates showed that acetylation of histones, assessed with a histone H3 pan-acetyl antibody, a histone H4 pan-acetyl antibody, and a H3K56ac specific antibody, is decreased by 25%-62% in *cra-1* mutants compared to wild type (Fig. 1D). This was further supported by the reduction in H3K56ac and H2AK5ac observed in whole mounted germlines of *cra-1* mutants compared to wild type (Fig. 1E-F). These observations suggest, first, that the pan acetylation antibody can reveal alterations in global histone acetylation, possibly because histones are a major component of the broader pool of lysine acetylated proteins identified by this reagent; and second, that CRA-1 has a role in modulating histone acetylation that is not restricted to a single lysine residue on histones, and instead affects global levels of histone acetylation.

Use of the pan acetylation antibody also revealed two interesting features regarding histone acetylation in wild type. First, an enrichment for acetylation foci on autosomes compared to the X chromosomes occurs during early prophase (transition zone to mid-pachytene; Fig. 1G). This suggests a higher level of histone acetylation on autosomes compared to the X chromosomes, which is consistent with both meiotic sex chromosome silencing during early prophase and the observations of autosomal enrichment for some specific histone acetylation markers [29,32,33]. Second, levels of histone acetylation change during germline progression (Fig. 1C). An overall decrease in histone acetylation is observed upon meiotic entry (transition zone), followed by a gradual increase as nuclei progress into the late pachytene stage. In *cra-1* mutant germlines, histone acetylation is reduced from the premeiotic tip to the late pachytene stage, with the most severe decrease detected during transition zone and pachytene stages compared to wild type. Taken together, these data identify a role for CRA-1 in regulating levels of global histone acetylation in the germline.

### CRA-1 localization supports its role as a positive regulator of global histone acetylation

**Fig 2:**
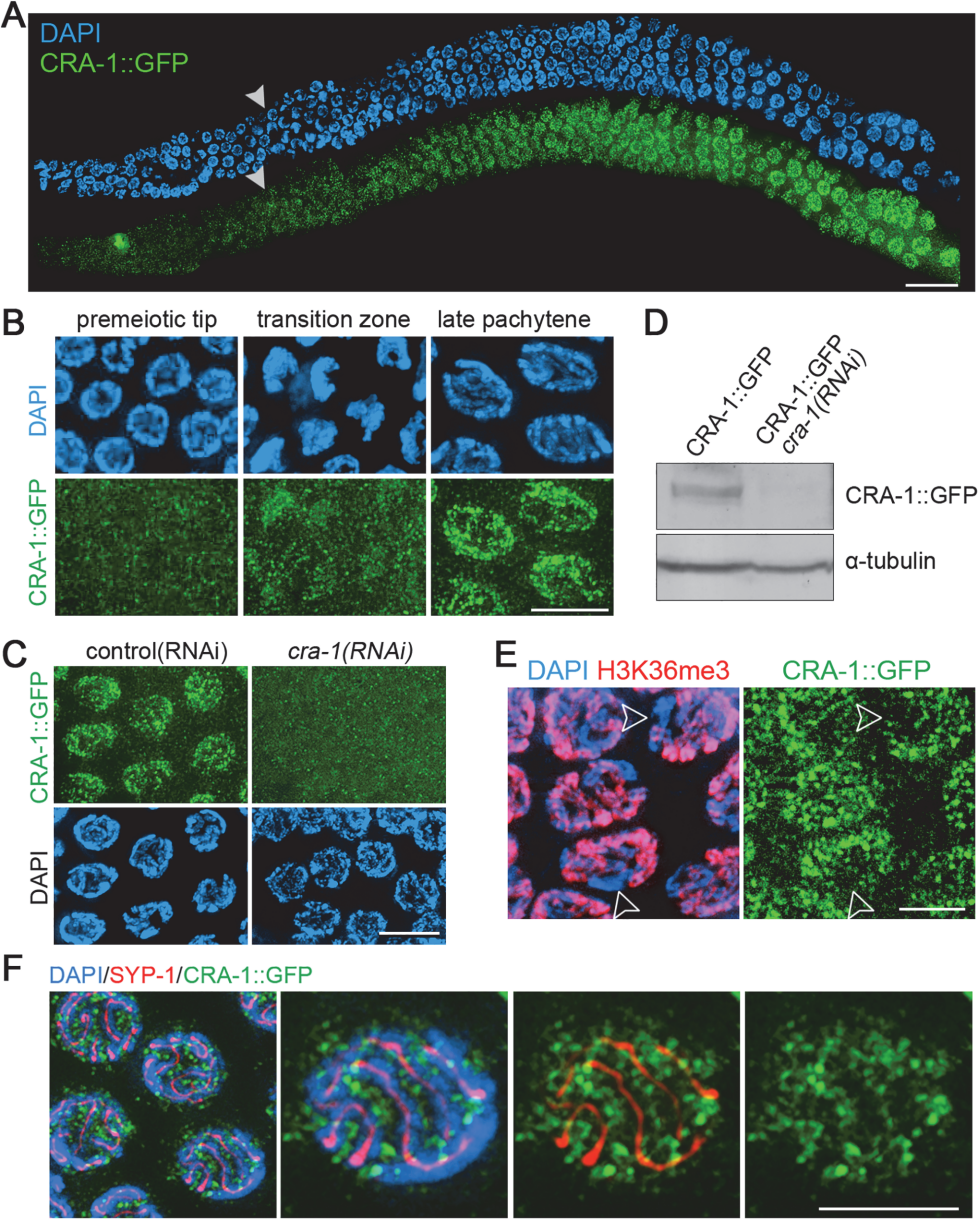
Expression and localization of CRA-1::GFP in the *C*. *elegans* germline. (A) CRA-1::GFP expression pattern in the adult hermaphrodite germline. Gonads dissected from CRA-1::GFP transgenic hermaphrodite worms were co-stained with anti-GFP antibody (green) and DAPI (blue). White arrowheads indicate entrance into meiosis (beginning of the transition zone). Bar, 20 μm. (B) High-magnification images of representative nuclei from different stages in the germline are shown to indicate CRA-1::GFP expression levels. Bar, 5 μm. (C) CRA-1::GFP expression in pachytene nuclei of gonads from either control(RNAi) (empty vector) or *cra-1(RNAi)* CRA-1::GFP transgenic worms. Gonads were stained with anti-GFP antibody (green) and DAPI (blue). Bar, 5 μm. (D) Western blot analysis of CRA-1::GFP and tubulin in control(RNAi) and *cra-1(RNAi)* CRA-1::GFP transgenic worms. (E) CRA-1::GFP is enriched on the autosomes. Pachytene nuclei were co-immunostained for CRA-1::GFP (green) and H3K36me3 (red). DNA was stained with DAPI (blue). Open arrowheads point to the X chromosomes. Bar, 4 μm. (F) Co-immunostaining for CRA-1::GFP (green) and SYP-1 (red) in pachytene nuclei of CRA-1::GFP transgenic worms. DNA was stained with DAPI (blue). Bar, 3 μm.

To further examine the link between changes in global histone acetylation and CRA-1 function we generated a transgenic line expressing a functional GFP tagged CRA-1 driven by a *cra-1* promoter (Figs. 2, S1, S2). CRA-1::GFP is observed localizing in somatic and embryonic cells as well as to meiotic germline nuclei (Figs. 2A-B, S2A and S2B), suggesting that the role of CRA-1 may not be limited to meiosis. This is consistent with the elevated levels of larval lethality (61%) observed in *cra-1* mutants [30]. The specificity of the observed CRA-1::GFP signal was confirmed by anti-GFP immunostaining of dissected gonads from transgenic worms depleted of CRA-1 by RNAi (Fig. 2C), and by western blotting (Fig. 2D). Analysis of CRA-1 localization during embryonic cell cycle progression does not show an obvious change of CRA-1 signal from interphase to mitotic prophase, although a signal reduction was observed from prometaphase to anaphase (S2C Fig.). In the germline, CRA-1 signal is first detected in early prophase nuclei (leptotene/zygotene stages), during which chromosomes reorganize spatially acquiring a crescent-shaped appearance (Fig. 2A-B). CRA-1 signal increases as meiotic nuclei progress into the pachytene stage, where chromosomes are fully synapsed and crossover formation is completed. Therefore, the impact of a *cra-1* mutation on histone acetylation along the gonad is consistent with the pattern of expression observed for CRA-1, supporting the idea that CRA-1 may contribute to this dynamic acetylation. Moreover, the CRA-1::GFP transgene can also restore histone acetylation in *cra-1* mutants (S1E Fig.). Therefore, these data are consistent with a role for CRA-1 as a positive regulator of global histone acetylation.

Immunostaining also revealed additional interesting features about CRA-1 localization. First, CRA-1 is enriched on autosomes, as indicated by the low levels of CRA-1::GFP signal on the X chromosome, identified via co-staining for H3K36me3, a histone modification that is tightly associated with active transcription and therefore enriched on the autosomes, but mostly absent on the X chromosome during early and mid prophase [34] (Fig. 2E). Second, although CRA-1 is required for SC assembly, it does not perform this function at the SC, since CRA-1::GFP does not localize to the interface of paired homologous chromosomes, where the SC is present, and instead exhibits a peri-chromosomal localization (Fig. 2F). Therefore, CRA-1 is an autosomally enriched protein, not a component of the SC, and its presence in both germline and somatic nuclei suggest its roles may extend beyond meiosis.

### The X chromosomes undergo lower levels of DSB formation compared to the autosomes

**Fig 3:**
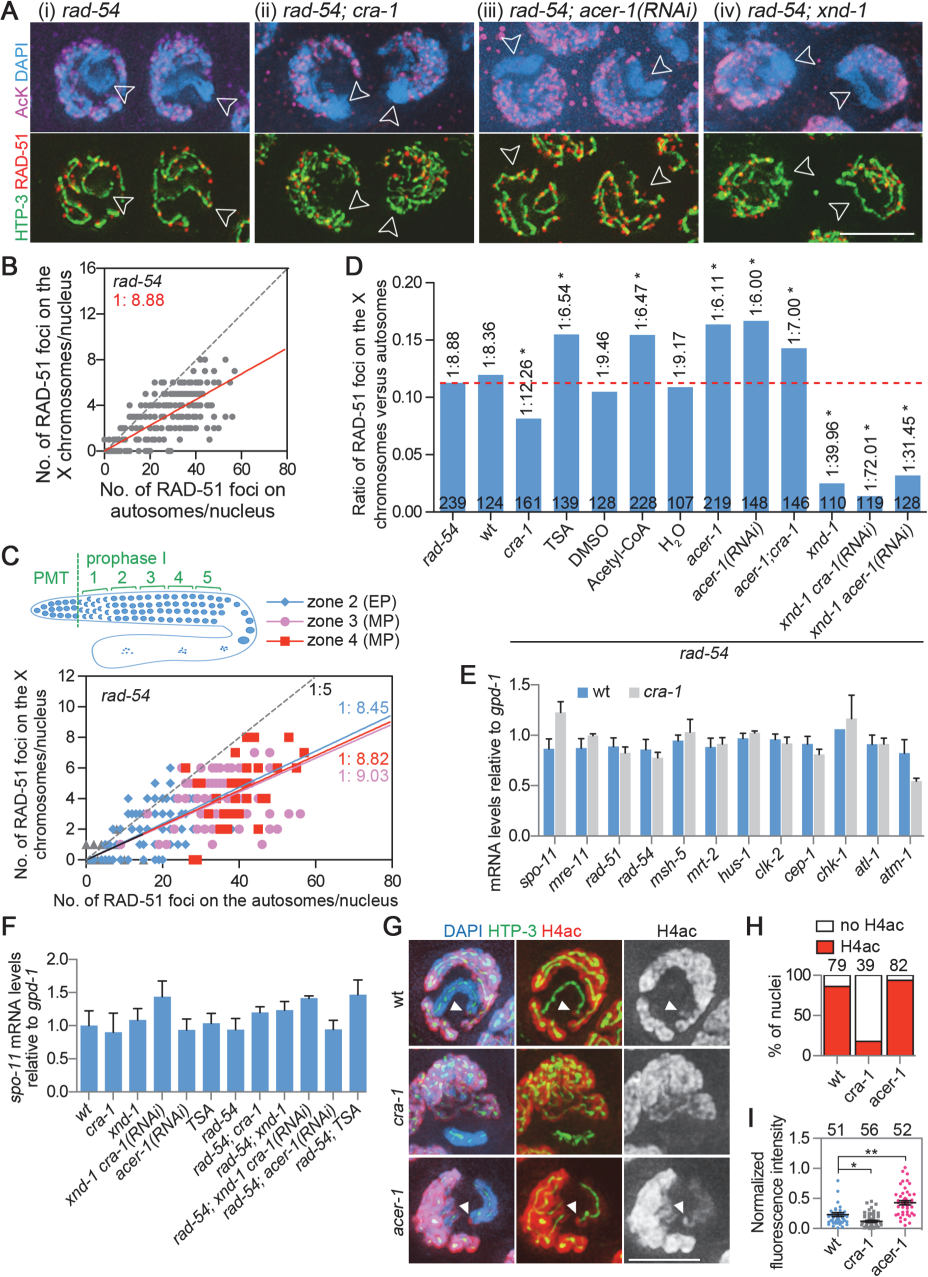
Analysis of DSB distribution, gene expression and histone acetylation. (A) Gonads from the indicated genotypes were co-immunostained for HTP-3 (green), RAD-51 (red) and AcK (magenta). DNA was stained with DAPI (blue). Images are projections halfway through 3D data stacks of whole nuclei. The X chromosomes show low levels of AcK staining during early meiotic prophase (indicated by open arrowheads). Bar, 5 μm. (B) Graph depicts the distribution of RAD-51 foci on the X chromosomes and the autosomes during early meiotic prophase (from transition zone to mid pachytene combined). The average ratio of RAD-51 foci on the X versus autosomes is indicated. Dashed lines indicate a X/A ratio of 1:5. (C) Graph depicts the distribution of RAD-51 foci on the X and the autosomes during early meiotic prophase (from zones 2 to 4, early through mid-pachytene). The average ratios of RAD-51 foci on the X versus autosomes are indicated. The dashed line indicates a X/A ratio of 1:5. (D) The average ratios of RAD-51 foci on the X versus autosomes in indicated genotypes or treatments in the *rad-54* mutant background. Data were analyzed as in (B). The numbers of nuclei scored for each genotype are indicated. Dashed red line depicts the ratio observed in *rad-54* single mutants. * P<0.0001 by the extra sum-of-squares F test, compared to *rad-54* single mutants. (E) Levels of *spo-11* and DSB repair gene expression in dissected gonads from wild type and *cra-1* mutants. Quantitative RT-PCR analysis revealed that the germline-specific gene expression for the depicted genes was not altered when comparing *cra-1* to wild type and normalizing to *gpd-1*. Experiments were performed as in [26], utilizing the primers described therein. Error bars represent SEM for three biological replicates, each performed in duplicate. (F) Levels of *spo-11* gene expression in indicated genotypes and treatment conditions. Quantitative RT-PCR was performed as in (E), except that whole worms were analyzed instead of dissected gonads. (G) Gonads from the indicated genotypes were co-immunostained for HTP-3 (green) and H4ac (red). DNA was stained with DAPI (blue). The white arrowheads indicate the H4ac-stained X chromosome tip. Bar, 3 μm. (H) Percentage of nuclei showing H4ac staining at the tip of the X chromosome in the indicated genotypes. The numbers of nuclei scored are indicated on the top. At least four gonads were scored for each genotype. (I) Average fluorescent intensity of H4ac along the X chromosomes and on the background was measured with Photoshop (Adobe systems Inc.) for each nucleus. The background fluorescent intensity was subtracted from the fluorescent intensity detected on the X chromosomes and this value was then normalized to the background fluorescent intensity. The numbers of nuclei measured are indicated on the top. At least four gonads were scored for each genotype. Bars represent the mean ± SEM. * P = 0.0006, ** P<0.0001, two-tailed Mann-Whitney test, 95% C.I.

To test for a biological significance of regulating global histone acetylation in the germline, we examined the distribution of DSBs between the X chromosomes and autosomes given their different chromatin states. To do this, we analyzed RAD-51 foci in *rad-54* mutants, where RAD-51, a protein required for strand invasion/exchange during DSB repair, associates with DSB repair sites, but DSB repair is blocked and DSB-bound RAD-51 are “trapped” and can be easily scored [24,25,35]. Therefore, RAD-51 foci are used herein as a surrogate for DSBs, with the caveat that this may not represent the total number of DSBs since we cannot discard the possibility that not all DSBs, particularly in mutant backgrounds, may be processed to load RAD-51. This analysis was coupled to co-immunostaining with the pan acetylation antibody, which allows for ease of identification of the X chromosomes, as they exhibit greatly decreased histone acetylation compared to the autosomes during early meiotic prophase (Fig. 1G; [32]), and an antibody against the HORMA domain-containing protein HTP-3 to trace chromosome axes and distinguish between DSBs on different chromosomes (Fig. 3A).

We found that the X chromosomes exhibit lower levels of DSB formation compared to the autosomes (Fig. 3Ai), with an average ratio of RAD-51 foci detected on the X chromosomes compared to the autosomes (X/A) of 1:9 (Fig. 3B). There are five pairs of autosomes and one pair of X chromosomes in a hermaphrodite meiotic prophase nucleus. The length of the X chromosome (∼17% of the genome) is close to the average length for each chromosome (i.e. 16.67% of the genome). Therefore, approximately 44% fewer DSBs are formed on the X chromosome pair compared to the average levels of DSBs generated on an autosomal pair. Moreover, comparison of the levels of RAD-51 foci between X chromosomes and autosomes in wild type also revealed a lower level of RAD-51 foci on the X chromosomes (1:8.36, Figs. 3D and S3ii). The low level of RAD-51 foci detected on the X chromosomes is not likely a result of an impenetrability to the antibody, since under the same fixation conditions, heterochromatin markers can be observed on the X chromosomes and on heterochromatic extrachromosomal arrays [36].

We extended this analysis to examine whether there are distinct time windows of DSB formation on the X chromosomes and autosomes. The window of gene expression silencing for the X chromosomes in hermaphrodite gonads was divided into four zones to score levels of RAD-51 foci on the X chromosomes and autosomes. This analysis revealed that there are no significant differences in the X/A ratios of RAD-51 foci from early to mid pachytene (zones 2 to 4) (Fig. 3C) (P = 0.5973, by Extra sum-of-squares F test), during which most of the meiotic RAD-51 foci are detected in wild type. Taken together, this analysis revealed that, although DSBs form with similar kinetics across all chromosomes, the levels of DSBs are lower in the highly heterochromatic X chromosomes compared to the autosomes.

### CRA-1 and normal levels of histone acetylation contribute to efficient DSB formation

The direct assessment of whether histone acetylation promotes DSB formation during meiosis in *C*. *elegans* will require the development of reagents and experimental approaches that are not currently available in this system. Specifically, recombination hotspots do not exist in this model, there are no SPO-11 antibodies or tagged SPO-11 lines, and approaches such as ChIP-Seq with the temporal resolution required for tracking early meiotic events in the multinuclear germline have not yet been established. However, to start to examine whether histone acetylation regulates DSB distribution between X chromosomes and autosomes, we utilized the triple immunostaining strategy described above and introduced the *rad-54* mutation into the *cra-1* mutant background where global histone acetylation is reduced. In *rad-54; cra-1* double mutants, levels of RAD-51 foci on the X chromosomes are reduced and a X/A ratio of 1:12 is observed (Figs. 3Aii, 3D, S3iii), corresponding to a 28% reduction in RAD-51 foci levels on the X chromosome compared to *rad-54* single mutants. This suggests histone acetylation may contribute to achieving normal levels of DSB formation on the X chromosomes. This idea is further supported by the increased levels of RAD-51 foci observed on the X chromosomes following either injection of Trichostatin A (TSA), an HDAC inhibitor that results in increased global histone acetylation (1:6.54; Figs. 3D, S3iv and S4A), or acetyl-CoA (1:6.47; Figs. 3D, S3vi and S4B). However, a comparison of the levels of RAD-51 foci detected on autosomes and X chromosomes revealed that while the effects of changes in global histone acetylation are more evident on the X chromosomes, the autosomes are also affected, albeit to a lesser degree (S4C–S4D Fig.). This difference may reflect the inherently distinct thresholds of histone acetylation between the X chromosomes and the autosomes during early meiotic prophase. Importantly, changes in the ratios of RAD-51 foci between the X and the autosomes parallel the changes observed in histone acetylation. This is consistent with alterations in the substrate (chromatin environment) for DSBs instead of in the DSB formation/repair machinery per se, otherwise levels of RAD-51 foci would have been impaired to the same extent in both the X and the autosomes. Indeed, the expression levels of *spo-11*, and of several other genes required for DSB repair and DNA damage response, are not altered in *cra-1* mutants (Fig. 3E). TSA or acetyl-CoA injections also did not affect the expression of *spo-11* (Fig. 3F). Moreover, histone acetylation on the generally silenced X chromosomes, assessed with the histone H4 pan-acetyl antibody, is further decreased in *cra-1* mutants compared to wild type (Fig. 3G-I). Taken together, our data suggests that histone acetylation may promote efficient DSB formation on both the X chromosomes and the autosomes.

### The timing of DSB formation is regulated in an XND-1-dependent and global histone acetylation-independent manner

**Fig 4:**
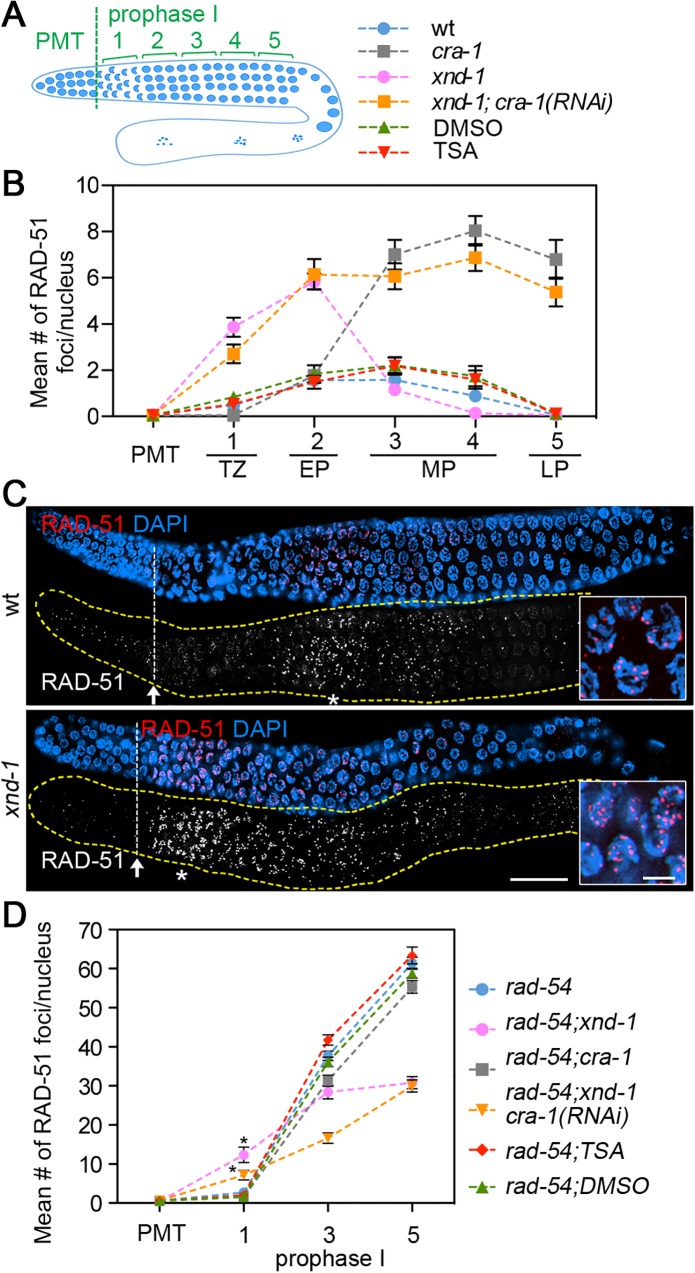
Time course analysis of DSB levels. (A) Diagram of a *C*. *elegans* germline showing the position of the zones scored for a time course analysis of RAD-51 foci levels. PMT (premeiotic tip); zones 1–5 correspond to: transition zone (1), early (2), mid (3–4), and late (5) pachytene stages of prophase I. (B) Time course analysis of RAD-51 foci levels in the indicated genotypes. Gonads were co-stained with an anti-RAD-51 antibody and DAPI. The mean number ± SEM of RAD-51 foci scored per nucleus is indicated for nuclei in the premeiotic tip (PMT) and five zones of meiotic prophase: TZ, transition zone; EP, early pachytene; MP, mid-pachytene; LP, late pachytene. (C) Gonads from wild type and *xnd-1* mutants were immunostained with an anti-RAD-51 antibody (red) and DNA was stained with DAPI (blue). Bar, 30 μm. Arrows show the position of meiotic entry. Asterisks show the position where levels of RAD-51 foci peak along the gonads in the different genotypes, and these regions are shown at a higher-magnification in the insets. Bar, 5 μm. (D) Graph shows the levels of RAD-51 foci observed along the germline in the indicated genotypes, all in a *rad-54* background where DSB-bound RAD-51 is “trapped”. Data represent mean number and SEM of RAD-51 foci per nucleus for premeiotic tip (PMT) and three zones of the germline. * P<0.001 by the two-tailed t test, 95% C.I.

We next assessed how changes in global histone acetylation might affect the timing of meiotic DSB formation. Immunostaining of *cra-1* mutant germlines had previously shown elevated levels of RAD-51 foci upon entrance into pachytene that remained elevated throughout late pachytene compared to wild type ([30]; Fig. 4A-B). Analysis of *xnd-1* mutants, where H2AK5ac is increased, revealed an increase in the levels of RAD-51 foci at transition zone and early pachytene (zones 1 and 2, respectively) compared to wild type (Fig. 4A-B). Moreover, levels of RAD-51 foci peaked at transition zone in *xnd-1* compared to early- to mid-pachytene in wild type (Fig. 4C). This observation suggests that DSBs might be formed earlier in *xnd-1* mutants compared to wild type, consistent with [37]. However, elevated levels of RAD-51 foci are still observed during early meiotic prophase (zones 1 and 2) in *xnd-1;cra-1(RNAi)* mutants. Moreover, TSA treatment does not affect the kinetics of RAD-51 foci along meiotic prophase compared to the control (Fig. 4B). These data indicate that changes in global histone acetylation do not affect the timing of DSB formation, which instead might be regulated by a separate function exerted by XND-1.

To assess how changes in global histone acetylation might affect the total number of DSBs formed during meiosis, a time course analysis of RAD-51 foci was performed for mutants in a *rad-54* background (Fig. 4D). Strikingly, DSB formation is significantly impaired in *rad-54; xnd-1* mutants, with the levels of RAD-51 foci at the late pachytene stage reduced by 50% compared to *rad-54* single mutants. The levels of RAD-51 foci are also reduced on the autosomes in *xnd-1* mutants (35% reduction at mid-pachytene, zone 4) compared to wild type (S4C Fig.). This data shows that XND-1 is required for efficient DSB formation on both the X chromosomes and the autosomes. Analysis of *rad-54; cra-1* double mutants and *rad-54; xnd-1; cra-1(RNAi)* triple mutants also revealed reductions in the levels of RAD-51 foci (e.g. 17% and 28%, respectively, at the mid-pachytene stage; Fig. 4D). Moreover, TSA injection increased the levels of RAD-51 foci during mid pachytene by 15% (P = 0.0026) and late pachytene by 8% (P = 0.077), compared to control *rad-54* worms (Fig. 4D). While the possibility that DSBs are shunted to a non-RAD-51-mediated repair pathway in these mutants cannot be completely eliminated, the simplest explanation for these data is that they indicate a change in DSB levels. Taken together, these data suggest that changes in global histone acetylation alter the levels, but not the timing, of meiotic DSB formation on both the X and the autosomes.

### Sites of early meiotic recombination events are located close to chromosome axes

**Fig 5:**
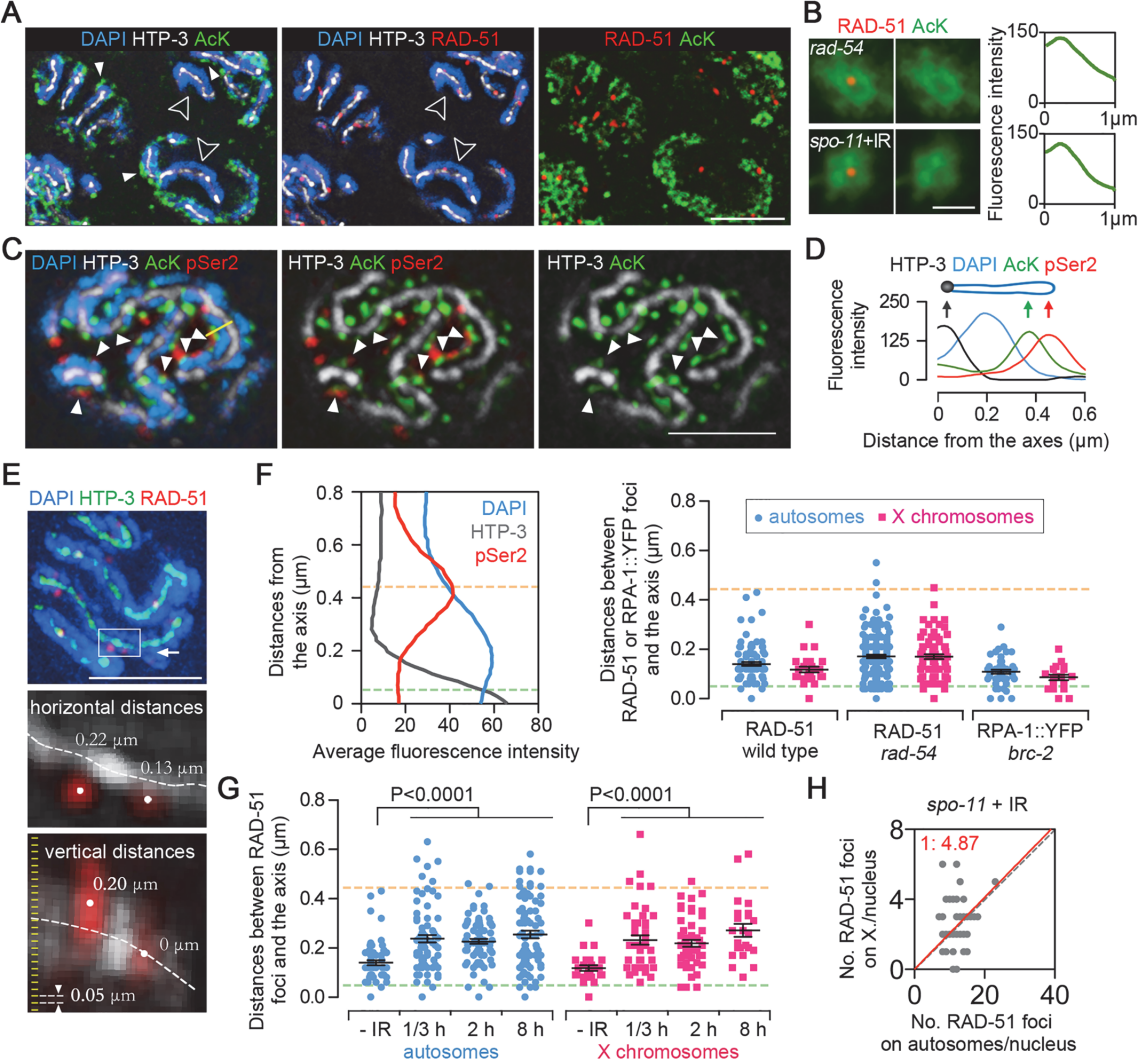
Sites of early meiotic recombination events are spatially separated from transcriptionally active chromatin. (A) Gonads from *rad-54* mutants were co-immunostained for HTP-3 (white), RAD-51 (red) and AcK (green, with a mouse anti-AcK antibody). DNA was stained with DAPI (blue). To facilitate visualization of individual chromosomes, images of pachytene nuclei correspond to 0.2 μm-thick projections from 3D data stacks consisting of 0.05 μm intervals. Bar, 4 μm. (B) Superimposition of at least 200 RAD-51 foci (red) captured from different nuclei from three different gonads at early pachytene in *rad-54* mutants or γ-IR treated *spo-11* mutants (20 min post-IR), showing that sites where DSB-bound RAD-51 foci are detected are devoid of AcK signal (green) (left panels). Average fluorescence intensity around the RAD-51 foci was measured by radial profile analysis (Image J) (right panels). Bar, 1 μm. (C) Co-immunostaining for CTD serine2-phosphorylated RNA polymerase II (pSer2) (red), AcK (green) and HTP-3 (white) in wild type worms. DNA was stained with DAPI (blue). To facilitate visualization of individual chromosomes, images of pachytene nuclei correspond to 0.2 μm-thick projections from 3D data stacks consisting of 0.05 μm intervals. Filled white arrowheads show examples where pSer2 and AcK are present at the same perichromosomal regions. Bar, 3 μm. (D) Fluoresence intensity was measured along the yellow line in (C) left panel, exemplifying how AcK and pSer2 are present at the tip of the chromatin loops. (E) Measuring the distances from RAD-51 foci to chromosome axes. Gonads were immunostained for RAD-51 (red) and HTP-3 (green). DNA was stained with DAPI (blue). Images were captured through whole nuclei at 0.05 μm intervals. Three-dimensional distances between the centers of RAD-51 foci and the centers of the axes (centers were defined by points of peak intensity) were measured with softWoRx Explorer (Applied Precision). Top image represents a stack of sections halfway through a whole nucleus. The horizontal and vertical distances from RAD-51 foci to the axes in the white box are shown at a higher resolution in the middle and bottom panels, respectively. Bar, 3 μm. (F) Left panel depicts the average fluorescence intensity of HTP-3 (gray line), DAPI (blue line) and pSer2 (red line) from the axis to chromosome periphery. Measurements were performed as in (D), and data represent average fluorescence intensity measurements for 41 germline nuclei from four different gonads. Yellow dashed line indicates the peak of pSer2 signal (tips of the chromatin loops). The HTP-3 signal corresponds to two parallel chromosome axes about 0.1 μm apart [30]. Thus, the axes are actually positioned about 0.05 μm from the center of the signal, depicted by a green dashed line (base of the chromatin loops). Right panel shows the distances between RAD-51 or RPA-1::YFP foci and chromosome axes. Gonads from wild type or *rad-54* mutant worms were triple-stained as in (A) and gonads from *brc-2;* RPA-1::YFP worms were immunostained for YFP, HTP-3 and AcK. 3D distances from the centers of the RAD-51 or RPA-1::YFP foci to the center of the axes were measured in early meiotic prophase as performed in (E). Dashed lines depict the positions indicated in the left panel. Bars represent the mean distances ± SEM. (G) Measurement of distances between γ-irradiation induced RAD-51 foci and axes. A dose of 800 rads was used to induce DSBs in *spo-11* mutants.—IR: analysis of early pachytene nuclei from *rad-54* mutants. The hours indicate the time points post-irradiation in which the worms were dissected for immunostaining. Dashed lines depict the positions indicated in (F). Bars represent the mean distances ± SEM. (H) Distribution of RAD-51 foci between the autosomes and X chromosomes in *spo-11* mutants exposed to 800 rads of γ-irradiation. Worms were dissected and fixed 20 min post-IR.

Our findings that meiotic DSBs are generated on the X chromosomes at lower levels (56%) compared to the autosomes, and that histone acetylation may promote DSB formation, are consistent with the idea that chromatin structure plays an important role in controlling DSB formation. Surprisingly, co-immunostaining of RAD-51 and acetylated lysine in *rad-54* mutants shows that RAD-51 foci do not colocalize with the strong acetylation foci during meiosis (Fig. 5A). Superimposition of 200 RAD-51 foci captured from different nuclei at early pachytene shows a greatly reduced AcK staining on the RAD-51 sites (Fig. 5B). Interestingly, both acetylation signals and an active transcription marker, CTD ser2-phosphorylated RNA polymerase II (pSer2), exhibit perichromosomal enrichment, flanking the DAPI signal and away from chromosome axes marked by HTP-3, suggesting that transcriptionally active genes may be localized at the tips of the chromatin loops (Fig. 5C-D). Therefore, our observation that RAD-51 foci do not co-localize with AcK suggests that DSB formation and/or the early stages of meiotic DSB repair take place close to chromosome axes. This is further supported by measurements of the distances between RAD-51 foci and the axes on autosomes and X chromosomes during early meiotic prophase (transition zone and early pachytene stages) (Fig. 5E). RAD-51 foci were observed in close proximity to chromosome axes during early meiotic prophase in both wild type and *rad-54* mutants. A similar result was obtained measuring distances of replication protein A (RPA), which binds to single-stranded DNA prior to RAD-51 following DSB end resection, in *brc-2* mutants (Fig. 5F). Importantly, this proximity to axes was not observed for RAD-51 foci resulting from γ-irradiation (γ-IR) induced DSBs in *spo-11* mutants (Fig. 5G), showing that the localization of RAD-51 foci close to chromosome axes is specific to endogenous DSB formation during early meiotic prophase. Furthermore, the distribution of DSBs generated by γ-IR on autosomes and X chromosomes, is also different from the distribution of endogenously produced DSBs. A X/A ratio of RAD-51 foci close to 1:5 is observed in irradiated-nuclei (P = 0.72) (Fig. 5H), indicating an even distribution of DSBs between the autosomes and X chromosomes.

Taken together, these data suggest that meiotic DSBs and/or early stages of DSB repair take place in close proximity to meiotic chromosome axes. This can be ascribed either to a “tethering” of loops to chromosome axes for DSB formation/repair as proposed in mouse and yeast [10–12] or to the formation of DSBs directly in regions of the loops very proximal to chromosome axes. We favor the former possibility, and note that although the RAD-51 foci did not co-localize with highly acetylated/transcribed regions, which our analysis suggests is located at the tips of the loops, DSB formation per se has been shown to induce chromatin silencing locally [38]. In fact, the γ-IR induced DSBs in *spo-11* mutants also exhibited reduced acetylation signal at the sites of RAD-51 foci (Fig. 5B). Thus, our data suggest that the “tethering” model and DSB induced chromatin silencing explain the reduced acetylation signal at RAD-51 sites. Further assessment of these chromatin features will require the development of new technologies for a high-resolution and stage-specific assessment of chromatin architecture and organization in the germline.

### CRA-1 interacts with and antagonizes ACER-1, a previously unknown and conserved acetyl-CoA hydrolase

**Fig 6:**
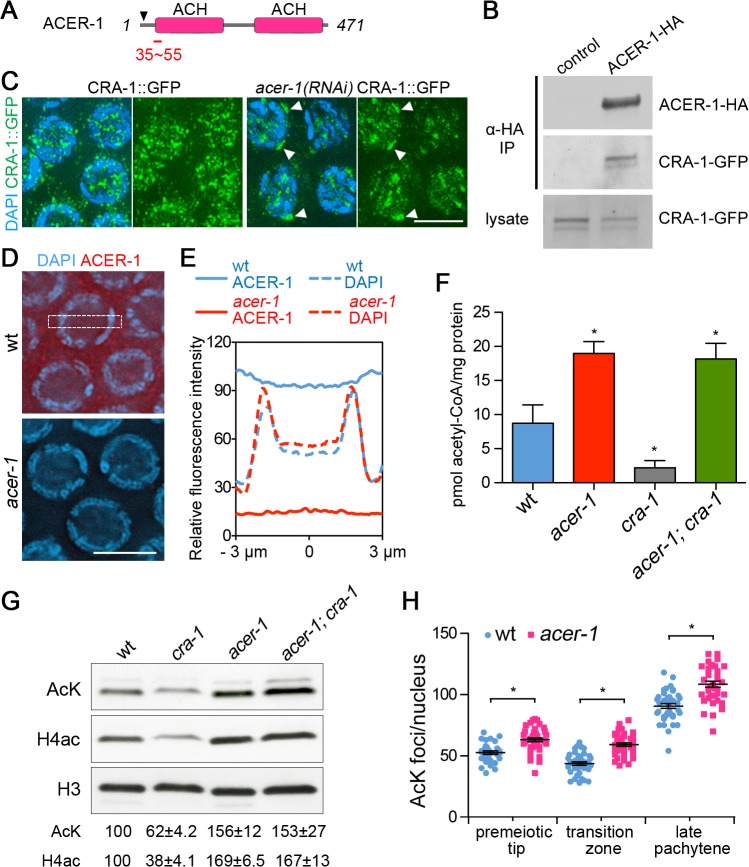
CRA-1 regulates histone acetylation by antagonizing ACER-1. (A) Schematic representation of the ACER-1 protein. ACER-1 contains two acetyl-CoA hydrolase (ACH) domains. Arrowhead indicates position of out-of-frame deletion. Red bar shows peptide region used for ACER-1 antibody production. (B) Vectors expressing CRA-1-GFP were cotransfected with ACER-1-HA or empty vectors (control) in 293T cells. The interaction of CRA-1-GFP with ACER-1-HA was analyzed by immunoprecipitation (IP) of the cell lysate with anti-HA agarose beads and Western blotting of the precipitate with anti-GFP antibody. (C) Co-staining with anti-GFP (green) and DAPI (blue) of pachytene nuclei from control and *acer-1(RNAi)* CRA-1::GFP transgenic worms. White arrowheads indicate CRA-1::GFP aggregates. Bar, 5 μm. (D) Immunolocalization of ACER-1 (red) in DAPI-stained (blue) pachytene nuclei of wild type and *acer-1* mutant worms. Images captured through the mid-section of nuclei were shown. Bar, 5 μm. (E) Measurement of ACER-1 immunostaining fluoresence intensity in the nucleus and cytoplasm. Fluoresence intensity was measured from images captured through the center of the nuclei in a rectangle area (1 μm x 6 μm) that covers both nucleus and cytoplasm for each cell with Image J. Data represent average signal measured from at least 20 nuclei from four different gonads for each genotype. (F) Measurement of acetyl-CoA in wild type and mutant worm lysates. * P<0.05 by the two-tailed t test, 95% C.I. (G) Western blot analysis of the global acetylation in wild type and mutant worm lysates detected with a pan acetylation antibody (AcK) and anti-H4ac antibody. The relative level of protein acetylation was determined by densitometric analysis of the western blot bands with ImageJ. Numbers represent mean ± SEM of data from at least three independent experiments. (H) Quantification of the number of acetylation foci observed per nucleus in germlines immunostained with anti-acetylated lysine antibody. Bars represent the mean number of foci ± SEM. * P<0.0001 by the two-tailed Mann-Whitney test, 95% C.I.

To understand the mechanism by which CRA-1 regulates global histone acetylation and meiotic DSB formation in *C*. *elegans*, we applied a proteomic approach to search for potential CRA-1 binding proteins. CRA-1::GFP was immunopurified from lysates of CRA-1::GFP transgenic worms and copurified proteins were indentified by mass spectrometry (see S1 Text). A list of identified proteins was generated following subtraction of proteins found in the control purification (S1 Table in S1 Text). While we did not identify a histone acetyltransferase by this approach, interestingly, we found ACER-1 (ORF C44B7.10) as a CRA-1 interacting protein. ACER-1 is a protein with homologs present from bacteria to humans (S5A Fig.) and it is a putative acetyl-CoA hydrolase/transferase (Fig. 6A). The interaction between CRA-1 and ACER-1 is further supported by co-expression in 293T cells, followed by immunoprecipitation and Western blot analysis (Fig. 6B). Moreover, depletion by RNAi of ACER-1 in CRA-1::GFP transgenic worms results in the aggregation of CRA-1::GFP in the nucleus (Figs. 6C, S5B), suggesting a direct relationship between CRA-1 and ACER-1 in *C*. *elegans*.

To determine how ACER-1 and CRA-1 function together to regulate acetyl-CoA, histone acetylation, and DSB formation, we first examined the localization of ACER-1. Immunostaining with an ACER-1-specific antibody revealed that ACER-1 localizes to both germline and somatic cells, but is germline-enriched (S6A–S6C Fig.), consistent with the enrichment of its mRNA detected in the germline (NextDB; http://nematode.lab.nig.ac.jp/db2/index.php). ACER-1 is present both in nuclei and the cytoplasm, showing a cytoplasmic enrichment at the premeiotic tip and early meiotic prophase, and an even distribution between the nucleus and the cytoplasm in late meiotic prophase and somatic cells (Figs. 6D-E, S6C-S6D). The presence of both ACER-1 and CRA-1 in the nucleus is consistent with the idea that these two proteins may interact in the nucleus to regulate histone acetylation levels. We next measured the levels of acetyl-CoA in worm lysates from wild type, *acer-1* and *cra-1* mutants. We found that the level of acetyl-CoA is significantly increased in *acer-1* mutants (P = 0.006 by two-tailed unpaired *t* test; Fig. 6F), indicating that ACER-1 acts as an acetyl-CoA hydrolase. Moreover, levels of acetyl-CoA are significantly reduced in *cra-1* mutants (P = 0.037), and are elevated in *acer-1;cra-1* double mutants, indicating that CRA-1 may antagonize ACER-1 acetyl-CoA hydrolase activity. Western blot analysis and immunostaining, using either the pan acetylation or histone H4 pan-acetyl antibodies, revealed that levels of histone acetylation are increased in both *acer-1* and *acer-1;cra-1* mutants, and reduced in *cra-1* mutants (Fig. 6G-H). These data suggest that CRA-1 can maintain the levels of acetyl-CoA in *C*. *elegans* by antagonizing ACER-1 activity, thus promoting global histone acetylation. Finally, levels of RAD-51 foci are increased by 32% and 24% on the X chromosomes in either *rad-54; acer-1* or *rad-54; acer-1; cra-1* mutants, respectively, compared to *rad-54* single mutants (X/A ratio is approximately 1:6 and 1:7, respectively; Fig. 3D, S3viii-x), consistent with the antagonistic functions of CRA-1 and ACER-1 in regulating histone acetylation. Importantly, increased histone acetylation is observed on the X chromosomes in *acer-1* mutants compared to wild type (Fig. 3I), and the increased levels of RAD-51 foci are not due to altered *spo-11* expression in *acer-1* mutants (Fig. 3F). Moreover, the transcriptional silencing of the X chromosomes during early meiotic prophase is not affected by the altered histone acetylation in both *cra-1* and *acer-1* mutants, as assessed by immunostaining of RNA polymerase II and its transcriptionally active form (pSer2), and by quantitative RT-PCR analysis of six germline-specific genes located throughout different regions of the X chromosomes (S7 Fig.). Therefore, these data further support a role for regulation of global histone acetylation in promoting DSB formation on the X chromosomes and uncover a link between metabolism and meiotic DSB formation.

### CRA-1, ACER-1 and XND-1 comprise a new protein network linking modulation of histone acetylation levels to meiotic DSB formation

**Fig 7:**
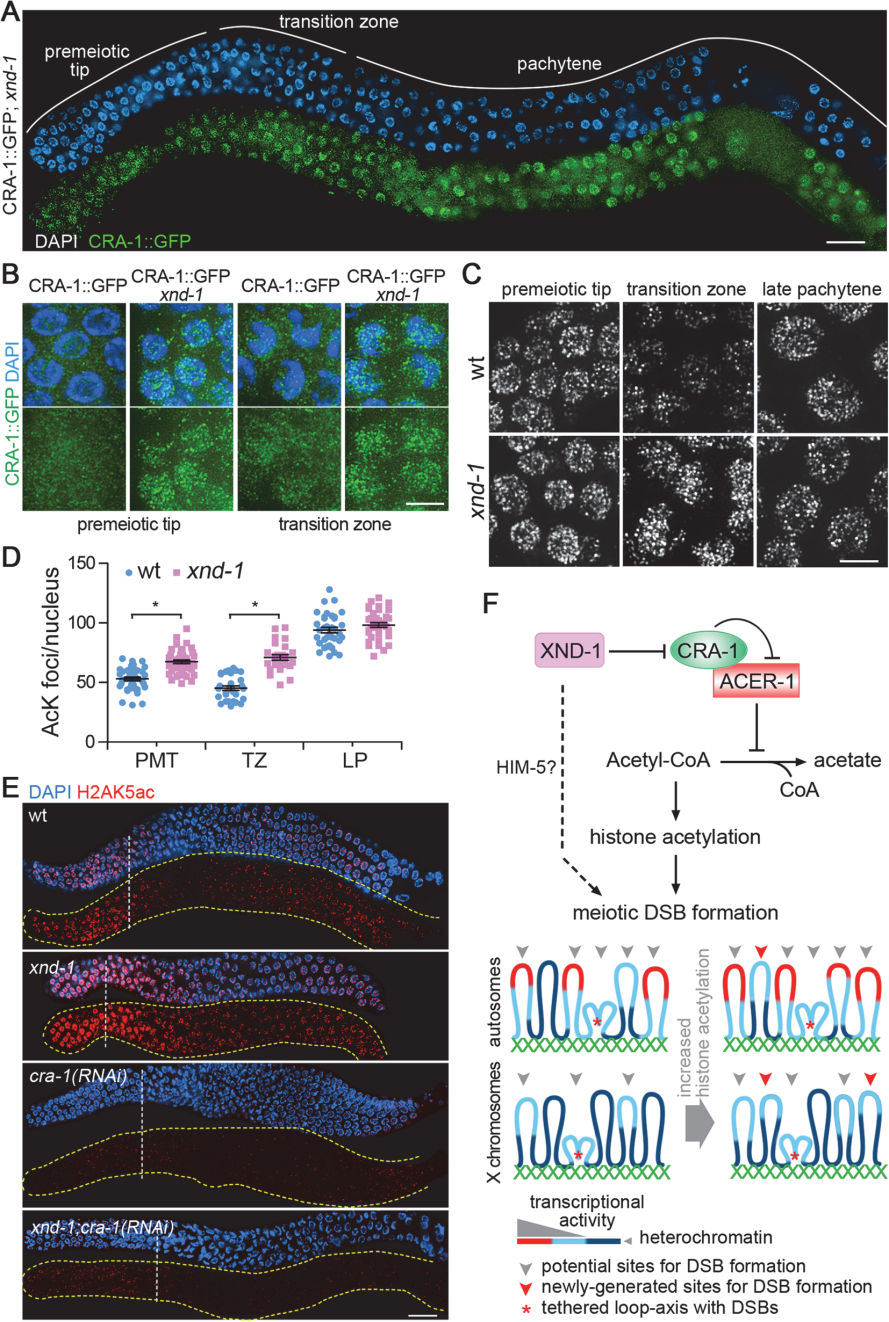
CRA-1 expression pattern is altered in *xnd-1* mutants. (A) CRA-1::GFP expression in *xnd-1* mutant gonads. Gonads dissected from CRA-1::GFP; *xnd-1* hermaphrodites were co-stained with anti-GFP antibody (green) and DAPI (blue). Bar, 20 μm. (B) High-magnification images of representative germline nuclei show the expression of CRA-1::GFP (green) at the premeiotic tip and transition zone in wild type and *xnd-1* mutants. Bar, 5 μm. (C) High-magnification images of nuclei at the indicated stages from wild type and *xnd-1* mutant gonads stained with an anti-acetylated lysine antibody. Images show projections through 3D data stacks of whole nuclei. Bar, 5 μm. (D) Quantification of the number of acetylation foci observed per nucleus in (D). Bars represent the mean number of foci ± SEM. * P<0.0001, by the two-tailed Mann-Whitney test, 95% C.I. (E) Gonads dissected from the indicated genotypes were co-stained with an anti-H2AK5ac antibody (red) and DAPI (blue). Bar, 20 μm. Yellow dashed lines were utilized to facilitate visualization of the gonad’s outline when only the antibody signal is depicted. White dashed vertical lines indicate exit from mitosis and entrance into meiosis. (F) A new histone acetylation-regulating protein network and sentinel chromosome model. In this regulatory network, XND-1 negatively regulates CRA-1, which interacts with and antagonizes ACER-1, an acetyl-CoA hydrolase, thereby providing a mechanism for regulating histone acetylation via modulation of acetyl-CoA levels. We propose that regulation of histone acetylation is linked to regulation of meiotic DSB formation. Dashed line: XND-1 promotes efficient meiotic DSB formation independent of its role in the histone acetylation regulatory network (potentially through HIM-5). This additional mechanism of function remains to be characterized. How are regulation of histone acetylation and meiotic DSB formation linked? Highly heterochromatic regions (dark blue) are not the preferred environments for DSB formation. Chromatin with a minimal level of histone acetylation, either transcriptionally active chromatin (red) or regions with lower histone acetylation thresholds (light blue), are better environments for DSBs. An increase in global histone acetylation does not remarkably increase the DSB-preferred chromatin regions on the autosomes, where they are already more prevalent due to their euchromatic state, but can significantly increase this type of site on the X chromosomes, where they are rare due to the highly heterochromatic environment. Our data suggests that DSBs and/or early stages of DSB repair take place close to chromosome axes. This could be either via tethering of loop sequences to axes, with DSB formation/repair taking place at axes as indicated by the red asterisk, or via breaks forming directly at sequences located near the base of the loops and in close proximity to axes, without any tethering (not shown). While we cannot exclude the latter, we favor the former given the evidence of chromatin silencing following DSB formation ([38]; Fig. 5B).

To further understand the mechanics by which CRA-1, ACER-1 and XND-1 regulate histone acetylation, and potentially meiotic DSB formation, we took advantage of the power of genetic and cytological analysis that can be combined in *C*. *elegans*. First, immunostaining with an anti-XND-1 antibody revealed that XND-1 expression and localization are not affected in *cra-1* mutants (S8 Fig.). However, the reciprocal experiment revealed that CRA-1::GFP expression was remarkably increased in both premeiotic tip and transition zone nuclei in *xnd-1* mutants compared to wild type (Fig. 7A-B). Second, altered dynamics of histone acetylation were also observed correlating with the altered CRA-1::GFP expression pattern in *xnd-1* mutants. While a reduction of acetylation levels was observed upon meiotic entry in wild type, no reduction was observed in *xnd-1* mutants (Fig. 7C). Quantification of acetylation foci showed that histone acetylation is increased in the *xnd-1* mutant both in premeiotic tip and transition zone nuclei compared to wild type (Fig. 7C-D) (P<0.0001), consistent with the altered pattern of expression observed for CRA-1 in *xnd-1* mutants. These data suggest that XND-1 acts upstream of CRA-1 to regulate histone acetylation. This is further confirmed by the analysis of H2AK5ac, previously shown to be increased in *xnd-1* mutants compared to wild type [29], but whose levels are reduced in both *cra-1(RNAi)* and *xnd-1; cra-1(RNAi)* worms (Fig. 7E). Therefore, XND-1 is responsible for the suppression of CRA-1 expression at the premeiotic tip and early stage of meiotic prophase, and loss of this suppression is accompanied by increased histone acetylation upon meiotic entry.

Interestingly, depletion of CRA-1 or ACER-1 in *rad-54;xnd-1* double mutants can either further reduce (X/A ratio of 1:72) or increase (1:31) the efficiency of DSB formation, respectively, on the X chromosome compared to *rad-54;xnd-1* mutants (1:40) (Figs. 3D, S3xi-xiii). These observations suggest that changes in global histone acetylation can still affect DSB formation on the X chromosomes in *xnd-1* mutants, supporting the idea that histone acetylation and XND-1 regulate DSB formation in different ways. In fact, HIM-5, a highly basic protein with no known orthologs outside *C*. *elegans* and no apparent effect on histone acetylation, is required for DSB formation on the X chromosomes, and chromosomal deposition of HIM-5 requires XND-1 activity suggesting that XND-1 might regulate DSB formation through HIM-5, by means yet to be determined [37]. Thus, although mutation of *xnd-1* promotes histone acetylation, it causes the loss of HIM-5 on the chromosomes, which results in a deficiency of DSB formation. Taken together, these studies place XND-1 as a negative regulator of CRA-1, which interacts with and antagonizes ACER-1, an acetyl-CoA hydrolase, thereby providing a mechanism for regulating histone acetylation via modulation of acetyl-CoA levels (Fig. 7F).

## Discussion

The frequencies and localization of meiotic DSBs on chromosomes can affect both the formation and the position of crossovers. However, the requirements of chromatin structure for DSB formation remained unclear. In this study, we identified a pathway for the regulation of global histone acetylation during meiotic prophase in *C*. *elegans*, and leveraged this discovery to find a connection between the levels of acetyl-CoA, histone acetylation and DSB formation on both the X and the autosomes, with stronger effects detected on the transcriptionally silenced X chromosomes. Our data are consistent with the idea that the early events of meiotic recombination occur near chromosome axes. Moreover, we uncovered a link between acetyl-CoA metabolism, meiotic DSB formation and the ensuing genetic diversity provided by meiotic DSB repair.

### Chromatin state controls DSB formation: the “Sentinel Chromosome” model

Meiotic DSBs are not uniformly distributed along the genome. There are discrete regions called hotspots that are preferred for DSB formation. However, it is still not clear how these discrete regions become “hot”. Chromatin state has been considered an important factor affecting DSB formation. H3K4me3, a modification that is enriched at gene promoter regions, has been suggested to play a conserved role in promoting DSB formation [12,15]. However, analysis of H3K4me3 enrichment and DSB formation in yeast showed little correlation [18]. In mice, levels of PRDM9-dependent H3K4me3 are much lower than PRDM9-independent H3K4me3 in promoter regions [17]. These observations suggest that additional modifications or factors might be required to direct DSB formation.

The distribution of DSBs along the genome at high-resolution is not known in *C*. *elegans*. However, the fact that the X chromosomes present a repressive chromatin environment where transcription is silenced during meiotic prophase provides a great opportunity to study the relationship between chromatin architecture/state and DSB formation. It has been previously suggested that a specific chromatin state is required for DSB formation, and that the timing of DSB formation might be different for X chromosomes and autosomes [39]. Here our data indicates that the timing of DSB formation is generally the same between the X chromosomes and the autosomes. DSB formation does not occur earlier on the X chromosomes. Interestingly, a lower frequency of DSB formation was observed on the X chromosomes, suggesting that the chromatin structure on autosomes is preferred for DSB formation. However, although the X chromosomes are silenced, DSBs still form at 56% of the level observed on the autosomes in *rad-54* mutants, indicating that the frequency of DSB formation does not highly correlate with the levels of transcription (i.e. chromatin state). Moreover, changes in global histone acetylation remarkably parallel the altered frequency of DSB formation on the X chromosomes. These and other data presented in this study lead us to propose a “sentinel chromosome” model in which the effects of altered histone acetylation on DSB formation may be detected more robustly on the X chromosomes due to the inherently lower levels of histone acetylation they exhibit in early prophase when DSB formation takes place. The X chromosome therefore behaves as the proverbial canary in a coal mine serving as a barometer of the alterations that can result from perturbing the regulation of histone acetylation. We propose that the sentinel chromosome model works as follows: DSB formation requires a minimal threshold of histone acetylation on the chromatin, which is still present in the generally silenced X chromosomes. However, a global increase in histone acetylation can promote an increase in the number of DSB sites in both autosomes and the X chromosomes, but while these are already more prevalent on autosomes due to their euchromatic state, they are rare in the highly heterochromatic X chromosomes. Therefore, this provides a mechanism in which changes in histone acetylation can differentially affect the distribution of DSBs on X chromosomes versus autosomes (Fig. 7F).

A deficiency in DSB formation on the X chromosomes has been observed in the context of various different mutants [29,37,39,40], which is consistent with the idea that the silenced X chromosomes face more challenges for DSB formation. The main barrier for DSB formation on the X chromosome might be the inaccessibility of its DNA for the recombination initiation machinery due to its highly heterochromatic architecture. Therefore, factors that promote an open chromatin state may be required for DSB formation on the X chromosomes more often than on the autosomes, due to the open property of the autosomes. Consistent with this, we found that an *xnd-1* mutation results not only in the absence of DSBs on the X chromosomes, but also a reduction of DSBs on the autosomes, although it remains to be examined whether XND-1 directly or indirectly regulates DSB formation (S4C–S4D Fig.).

### Acetyl-CoA connects metabolism with genomic diversity

Acetyl-CoA is an important metabolic product and is used for the generation of citrate, an intermediate of the tricarboxylic acid (TCA) cycle, which generates energy from carbohydrates, fats and proteins in mitochondria. Because acetyl-CoA cannot be directly transferred through the membrane, there are distinct pools of acetyl-CoA in the cells: a mitochondrial pool and a nucleocytoplasmic pool. Acetylation of histones and many other proteins depends on the nucleocytoplasmic pool of acetyl-CoA. In mammals, acetyl-CoA can be transferred from the mitochondria to the cytoplasm in the form of citrate through ATP-citrate lyase (ACL). It has been shown that under high nutrient conditions, acetyl-CoA produced by the ACL is the predominant source for histone acetylation [41]. In *C*. *elegans*, there are homologs of mammalian ACL, so the mitochondrial acetyl-CoA most likely can be transferred to the cytoplasm, albeit this remains to be shown. In this study, we detected the combined pools of acetyl-CoA, and we found that the total level of acetyl-CoA is altered in *cra-1* and *acer-1* mutants. Based on the observation that both CRA-1 and ACER-1 are present in the nucleus and that depletion of ACER-1 causes aggregation of CRA-1 in the nucleus, we would expect that CRA-1 and ACER-1 might directly regulate the nucleocytoplasmic pool of acetyl-CoA.

Acetyl-CoA production is nutrient-dependent. In proliferating cells, glucose and glutamine are the primary carbon sources that contribute to the production of citrate. Excess citrate can be used for the generation of nucleocytoplasmic acetyl-CoA, which in turn promotes histone acetylation and gene transcription [42]. Acetyl-CoA thus acts as a key sensor of metabolic states and controls gene transcription. However, in multicellular organisms, cells are differentiated with specific functions, and metabolic state might not necessarily be associated with gene transcription. This requires fine regulation of the nucleocytoplasmic acetyl-CoA level. Therefore, the interaction between CRA-1 and ACER-1 may act as an important control of acetyl-CoA and histone acetylation.

In this study we showed a link between the levels of acetyl-CoA, histone acetylation and DSB formation on chromosomes, with a remarkable impact on the X chromosomes. Although the effects are weaker on the autosomes, a shift of DSB-preferred sites might be induced. The increase and shift of DSB sites may provide more potential sites for COs, thus promoting genetic diversity among the corresponding offspring. This therefore supports a connection between metabolic state and the genetic diversity that stems from the DSB-dependent meiotic CO exchanges. Interestingly, it has been shown that under stress conditions, worms that normally reproduce through self-fertilization can increase the generation of male animals, as well as their outcrossing ability, to promote adaptation [43]. Thus, low frequencies of DSB formation on the X chromosomes under low nutrient conditions may result in increased male frequencies providing for the beneficial outcome of increased outcrossing.

In summary, our study identified a pathway for the regulation of global histone acetylation by modulating the levels of acetyl-CoA during meiotic prophase in *C*. *elegans*. Moreover, we link elevated levels of acetyl-CoA and histone acetylation to the promotion of DSB formation especially on the X chromosomes. Thus, we uncovered the underpinnings of a key mechanism that we propose is set in place to bypass the highly heterochromatic state of the X chromosomes ensuring they successfully undergo DSBs during meiosis, and establishing a connection between acetyl-CoA metabolism and genomic diversity.

## Materials and Methods

### *C*. *elegans* genetics

N2 Bristol was used as the wild type strain. To generate CRA-1::GFP transgenic worms, a CRA-1::GFP construct containing the *cra-1* promoter, *cra-1* genomic sequence and a *gfp* sequence was integrated by the MosSCI (Mos1-mediated single copy gene insertion) transposon-mediated insertion technique into the ttTi5605 site on chromosome II, as described in [44]. To confirm that the GFP fusion construct is functional and its localization reflects endogenous CRA-1 localization, we introduced the CRA-1::GFP transgene into *cra-1* mutants. The CRA-1::GFP transgene rescued the synapsis defects of *cra-1* mutants, and COs were restored given that six bivalents (pairs of DAPI-stained chromosomes attached through chiasmata) were observed instead of the 11 to 12 univalents characteristic of *cra-1* mutants (S1A–S1B Fig.). Moreover, brood size and embryonic lethality were significantly, albeit not completely, restored in the rescued line (S1C–S1D Fig.). This partial rescue suggests that the GFP tag may affect other functions played by CRA-1. *acer-1(rj15)* mutants were generated via CRISPR-Cas9 technology as described in [45]. The guide RNA sequence targets a site near the start codon: ATGCTTTCTCGGCTTACATCTCGCTCCCTCGGAACCTCGGCTGCGTGCTCAAGA. The underlined sequence is deleted in the *acer-1* mutant resulting in an out-of-frame deletion.

All worms were cultured at 20^°^C under standard conditions as described in Brenner (1974). The following mutations and chromosome rearrangements were used: LG I: *rad-54(ok615)*; LG II: *acer-1(rj15)*; LG III: *brc-2(tm1086)*, *xnd-1(ok709)*, *cra-1(tm2144)*; LG IV: *spo-11(ok79)*; and LG V: *syp-1(me17)* [8,25,29,30,46]. Transgenes: *opIs263[Prpa-1*::*RPA-1-YFP*::*3’-URR]* [47].

### Immunofluorescence microscopy

Immunostaining was performed as in [30]. Antibodies were used at the following dilutions: rabbit α-SYP-1 (1:200; [46]), guinea pig α-HIM-8 (1:200; [48]), rabbit α-ACER-1 (1:10000), guinea pig α-HTP-3 (1:300; [49]), guinea pig α-XND-1 (1:500; [29]), rabbit α-RAD-51 (SDIX, 1:20000), mouse α-pSer2 (H5) (Covance, 1:100), rabbit α-AcK (Cell Signaling Technology, 1:10000), mouse α-AcK (Cell Signaling Technology, 1:1000), rabbit α-H3K36me3 (1:300), rabbit α-H3K56ac (Millipore, 1:500), rabbit α-H2AK5ac (Cell Signaling Technology, 1:400), chicken α-GFP (Abcam, 1:400), rabbit α-H4ac (Active motif, 1:1000), and mouse α-tubulin (Sigma, 1:500). The pan acetylation antibody was utilized both at a high (1:10,000) and low (1:1,000) dilutions. While the latter exhibited a broad distribution over chromatin, it did not allow for precise quantification of changes in signal intensity, possibly due to saturation. The 1:10,000 dilution was therefore utilized for quantification. The following peptide sequence was utilized for ACER-1 antibody production in rabbits: CGKSPKVVSLAEATRDIKSGDN, and antibody specificity was confirmed by immunostaining and western blot analysis (S6A–S6D Fig.). The following secondary antibodies from Jackson ImmunoResearch Laboratories were used at a 1:200 dilution: α-chicken FITC, α-rabbit Cy3, α-rabbit FITC, α-mouse FITC, α-mouse Cy5 and α-guinea pig FITC. DAPI (Sigma, 1 μg/ml) was used to counterstain DNA. Images presented are either partial or whole projections through 3D data stacks of nuclei. Immunofluorescence images were captured through whole nuclei at 0.2 μm or 0.05 μm intervals with an IX-70 microscope (Olympus) and a cooled CCD camera (CH350; Roper Scientific) under the control of the DeltaVision system with SoftWoRx software (Applied Precision) and deconvolved using a conservative algorithm with 15 iterations.

### Western blot

Whole worm lysates were prepared from young adult worms (24 hours post-L4) by freezing and then boiling the worms in Laemmli sample buffer. Proteins were resolved by SDS-PAGE and then transferred onto polyvinylidene difluoride membranes (Millipore, 0.2 μm pore size) in transfer buffer (25 mM Tris-HCl pH 8.3, 192 mM glycine, 20% (v/v) methanol and 0.1% (w/v) SDS). The presence of SDS ensures histones can be completely transferred onto the membrane. Antibodies were used at the following dilutions: chicken α-GFP (Abcam, 1:2000), mouse α-HA (Sigma, 1:2000), rabbit α-AcK (1:3000), rabbit α-H3ac (Active motif, 1:3000), rabbit α-H4ac (Active motif, 1:3000), rabbit α-H3 (Cell signaling, 1:5000), rabbit α-H3K56ac (Millipore, 1:3000), mouse α-tubulin (Sigma, 1:2000), rabbit α-ACER-1(1:3000). Horseradish peroxidase-conjugated secondary antibodies from Jackson ImmunoResearch Laboratories were used at a 1:5000 dilution. Specific proteins were visualized with Pierce ECL Western Blotting Substrate. The relative level of acetylated histones was determined by densitometric analysis of the western blot bands (AcK relative to H3) using ImageJ software.

### Quantification of the number of acetylation foci

To compare the number of acetylation foci between germlines from different genotypes, gonads from every two genotypes were fixed on a single slide, either mixed together if the changes in chromosome morphology permit ease of identification of the genotypes, or fixed in separate areas of the same slide when chromosome morphology is indistinguishable between genotypes. Immunostaining was performed as described above. Images were captured and processed under the same exact conditions. At least five gonads were quantified per genotype. The average number of nuclei scored per zone for a given genotype ± standard deviation were as follow: mitotic tip, n = 56 ± 11; transition zone, n = 51 ± 8; late pachytene, n = 67 ± 13.

### Time course analysis for RAD-51 foci

Quantification of RAD-51 foci along the germline was performed as in [24]. Immunostaining of *C*. *elegans* germlines was performed in age-matched (24 h post-L4) animals as described above. At least four gonads were quantified per genotype. The average number of nuclei scored per zone for a given genotype ± standard deviation were as follow: mitotic tip, n = 112 ± 13; meiotic prophase zone 1 (transition zone), n = 92 ± 10; zone 2 (early pachytene), n = 108 ± 19; zone 3 (mid-pachytene), n = 78 ± 9; zone 4 (mid-pachytene), n = 70 ± 5; zone 5 (late pachytene), n = 63 ± 4.

### TSA and Acetyl-CoA injection

Worms were injected at 6 h post-L4 stage, and the injected worms were fixed and immunostained at 18 hours post injection. For Trichostatin A (TSA, Sigma) treatment, a concentration of 10 μM TSA was used and 0.2% DMSO (v/v) was injected as control. For Acetyl-CoA (Sigma) injection, a concentration of 100 μM Acetyl-CoA was used, and H_2_O was injected as control.

### CRA-1::GFP IP for mass spectrometry

CRA-1::GFP transgenic worms and N2 worms were lysed by vortexing in 15ml conical tubes containing lysis buffer (50 mM HEPES pH7.4; 1 mM EGTA; 3 mM MgCl2; 300 mM KCl; 10% Glycerol; 1% NP-40; 1 mM DTT; protease inhibitor mixture (Roche)) and small shards of broken glass cover slips. CRA-1::GFP and its binding proteins were immunopurified from the CRA-1::GFP worm lysates by using anti-GFP agarose beads (MBL International) with 2 hours incubation at 4^°^C. After being washed with lysis buffer, the beads were eluted with Glycine buffer (0.1 M pH2.5). The control purification was performed by using N2 worm lysates and the same anti-GFP agarose beads. Proteins eluted from the beads were precipitated with 20% trichloroacetic acid (TCA), and the resulting pellet was washed once with 10% TCA and four times with cold acetone.

### Acetyl-CoA quantification

Worms were washed twice in M9 buffer and three times in worm lysis buffer (50 mM KCl; 10 mM Tris pH5; 2.5 mM MgCl_2_; 0.45% NP-40; 0.45% Tween 20; 0.01% gelatin; 0.2 μg/μl proteinase K), and then frozen in liquid nitrogen. Worms were then incubated at 60^°^C for 2 hours. Proteinase K was inactivated with heating at 95^°^C for 20 minutes. The lysates were centrifuged for 20 minutes at 12,000 rpm and supernatants were used for measuring Acetyl-CoA and either protein or peptide concentration. Levels of Acetyl-CoA were measured with the PicoProbe Acetyl-CoA Assay Kit (BioVision) following the manufacturer’s instructions.

### RNAi

RNAi by microinjection was performed to deplete ACER-1. Double stranded RNA was produced by *in vitro* transcription (Ambion) and was injected into the gonads of young adult worms (12 h post-L4) at a concentration of 1 μg/μl. The *acer-1(RNAi)* worms examined consisted of young adults (24h post-L4) from the F1 generation. Single worm RT-PCR detection shows that *acer-1* expression is only partially suppressed following RNAi depletion (S5B Fig.). Feeding RNAi was used for depletion of CRA-1 as described in [50]. CRA-1 cDNA was cloned into the pL4440 feeding vector. Control RNAi was performed by feeding worms with HT115 bacteria carrying the empty pL4440 vector.

## Supporting Information

S1 Fig Assessing rescue of *cra-1* mutant phenotypes by GFP-tagged CRA-1. (A) Defects in synapsis and crossover formation in *cra-1* mutants. Gonads from *cra-1* mutant worms were immunostained for the axial and central region components of the SC, HTP-3 (green) and SYP-1 (red), respectively. DNA was stained with DAPI. Nuclei show discontinuous stretches of SYP-1 staining indicating unsynapsed chromosomes. 11 to 12 DAPI-stained bodies are observed in oocytes at diakinesis indicating lack of chiasmata. Bar, 5 μm. (B) CRA-1::GFP rescues the synapsis defects observed in *cra-1* mutants. Gonads dissected from CRA-1::GFP; *cra-1* worms were co-stained with anti-HTP-3 (green), anti-SYP-1 (red) and DAPI (blue). Insets: left and middle show pachytene nuclei with normal synapsis; right, six DAPI-stained bodies can be observed in the -1 oocyte corresponding to the six pairs of attached homologous chromosomes in *C*. *elegans* hermaphrodites. Bar, 5 μm. (C) Brood size is significantly increased in the CRA-1::GFP; *cra-1* line compared to *cra-1* mutants. * P<0.0001, two-tailed Mann-Whitney test, 95% C.I. (D) Embryonic lethality is reduced among the offspring of the CRA-1::GFP; *cra-1* line compared to *cra-1* mutants. * P<0.0001, two-tailed Mann-Whitney test, 95% C.I. (E) Global histone acetylation is rescued in the CRA-1::GFP; *cra-1* line compared to *cra-1* mutants. Anti-acetylated lysine antibody (AcK) was used to detect global histone acetylation. The levels of histone H3 and α-tubulin were used as loading controls. The relative level of acetylated histones was determined by densitometric analysis of the western blot bands (AcK vs. H3) using ImageJ. Numbers represent mean ± SEM for data from at least two independent experiments.
(TIF)

S2 Fig CRA-1 expression in embryonic and somatic cells. (A) Co-staining with an anti-GFP antibody (green) and DAPI (blue) in embryos from CRA-1::GFP transgenic adult worms. Bar, 5 μm. (B) Co-staining with an anti-GFP antibody (green) and DAPI (blue) of an intestinal nucleus from CRA-1::GFP transgenic adult worms. Bar, 5 μm. (C) CRA-1::GFP expression during embryonic cell cycle progression. CRA-1::GFP embryos were immunostained with anti-GFP antibody (green) and anti-α-tubulin antibody (red). DNA (blue) was stained with DAPI. Bar, 5 μm.
(TIF)

S3 Fig Analysis of DSB distribution. Graphs depict the distribution of RAD-51 foci levels detected on the X chromosomes and the autosomes during early meiotic prophase (from transition zone to mid pachytene all combined). The average ratios of DSBs inferred from the quantification of RAD-51 foci on the X versus autosomes are indicated. Dashed lines indicate a X/A ratio of 1:5.
(TIF)

S4 Fig Comparing the timing and levels of DSB formation on the X chromosomes and the autosomes. (A) Gonads from wild type worms injected with 10 μM TSA or 0.2% DMSO (v/v) were immunostained with a pan acetylation antibody (red) and DNA was stained with DAPI (blue). Shown are late pachytene nuclei. Bar, 5 μm. (B) Gonads from wild type worms injected with H_2_O or 100μM Acetyl-CoA were immunostained with a pan acetylation antibody (red) and DNA was stained with DAPI (blue). Shown are late pachytene nuclei. Bar, 5 μm. (C) Analysis of RAD-51 foci levels on autosomes in mid pachytene (zone 4) nuclei in the indicated genotypes. Bars represent the mean number ± SEM of RAD-51 foci observed on autosomes per nucleus. The fold changes in the mean numbers of RAD-51 foci on autosomes relative to *rad-54* single mutants are indicated for each genotype (red numbers). * P≤0.0077, two-tailed Mann-Whitney test, 95% C.I. (D) Analysis of RAD-51 foci levels on the X chromosomes in mid pachytene (zone 4) nuclei in the indicated genotypes. Bars represent the mean number ± SEM of RAD-51 foci observed on the X chromosomes per nucleus. The fold changes in the mean numbers of RAD-51 foci on the X chromosomes relative to *rad-54* single mutants are indicated for each genotype (red numbers). * P≤0.0155.
(TIF)

S5 Fig ACER-1 homologs and ACER-1 depletion by RNAi. (A) ACER-1 homologs present from bacteria to humans. Homologs were identified through a HHPRED search, which is based on similarity both in sequences and structure. The acetyl-CoA hydrolase and CoA-transferase domains share a high degree of similarity in both sequence and structure, consistent with previous findings that an acetyl-CoA hydrolase domain may have both hydrolase and transferase activity [51,52]. (B) Single-worm RT-PCR analysis of *acer-1(RNAi)* worms. Depletion of ACER-1 was obtained by microinjection of *acer-1* dsRNA. Single worm RT-PCR was performed to analyze RNAi efficiency comparing control (empty vector) worms to *acer-1(RNAi)* worms. *cep-1/p53* was assessed as a loading control.
(TIF)

S6 Fig ACER-1 is expressed in both germline and somatic cells. (A) Co-staining of wild type and *acer-1* mutant germlines with an anti-ACER-1 antibody (red) and DAPI (blue). Gonads from wild type and *acer-1* mutants were fixed and immunostained on the same slides. All images were captured under the same exposure conditions with the DeltaVision system (Applied Precision). Yellow dashed lines were utilized to facilitate visualization of the gonad’s outline when only the antibody signal is depicted. Bar, 30 μm. (B) Western blot analysis of ACER-1 and histone H3 in whole worm lysates from wild type, *cra-1* and *acer-1* mutants. (C) Image shows ACER-1 (red) immunostaining in wild type germline (diakinesis) and intestine. DNA was stained with DAPI (blue). Bar, 10 μm. (D) Co-staining of wild type and *acer-1* mutant intestinal cells with an anti-ACER-1 antibody (red) and DAPI (blue). Bar, 5 μm.
(TIF)

S7 Fig Analysis of transcriptional activity on the X chromosomes. (A) Co-staining with anti-RNA polymerase II antibody (Covance, clone CTD4H8, 1:200) (red) and DAPI (blue) of early pachytene nuclei from the indicated genotypes. Open arrowheads indicate X chromosomes. The pattern of RNA Polymerase II localization between autosomes and the X chromosomes is not altered in *cra-1* and *acer-1* mutants compared to wild type. Bar, 3 μm. (B) Co-staining with pSer2 antibody (Covance, clone H5, 1:100) (red) and DAPI (blue) of early pachytene nuclei from the indicated genotypes. Open arrowheads indicate X chromosomes. The localization pattern of pSer2 between autosomes and the X chromosomes is not altered in *cra-1* and *acer-1* mutants compared to wild type. Bar, 3 μm. (C) Expression levels of six genes on the X chromosome from dissected gonads of wild type and *cra-1* mutants. Quantitative RT-PCR analysis revealed that the germline-specific gene expression for the depicted genes was not altered when comparing *cra-1* to wild type and normalizing to *gpd-1*. (D) Analysis was performed as in (C) except that *acer-1* mutants were used instead of *cra-1* mutants. Expression levels of the six genes were not altered in *acer-1* mutants compared to wild type.
(TIF)

S8 Fig Immunostaining of XND-1 in wild type, *cra-1* and *xnd-1* mutant germlines. Gonads dissected from wild type, *cra-1* and *xnd-1* mutants were immunostained with an anti-XND-1 antibody. Gonads are oriented such that progression through meiosis is from left to right. White arrowheads indicate entrance into meiosis (beginning of the transition zone). Bar, 20 μm.
(TIF)

S1 Text Protocol for Mass Spectrometry and potential CRA-1 interacting proteins. Immunoprecipitation from CRA-1::GFP whole worm lysates with an anti-GFP antibody was analyzed by mass spectrometry. Experiment was performed in triplicate. The potential CRA-1 interacting proteins that were identified in at least two of the experiments were listed. Numbers indicate the total mass spectra collected from 3 experiments.
(DOCX)
