# Supplementary figures and images for: NatB Domain-Containing CRA-1 Antagonizes Hydrolase ACER-1 Linking Acetyl-CoA Metabolism to the Initiation of Recombination during C. elegans Meiosis
Source: PLoS Genet. 2015 Mar 13;11(3):e1005029. doi: 10.1371/journal.pgen.1005029 (PMC4359108; doi:10.1371/journal.pgen.1005029)

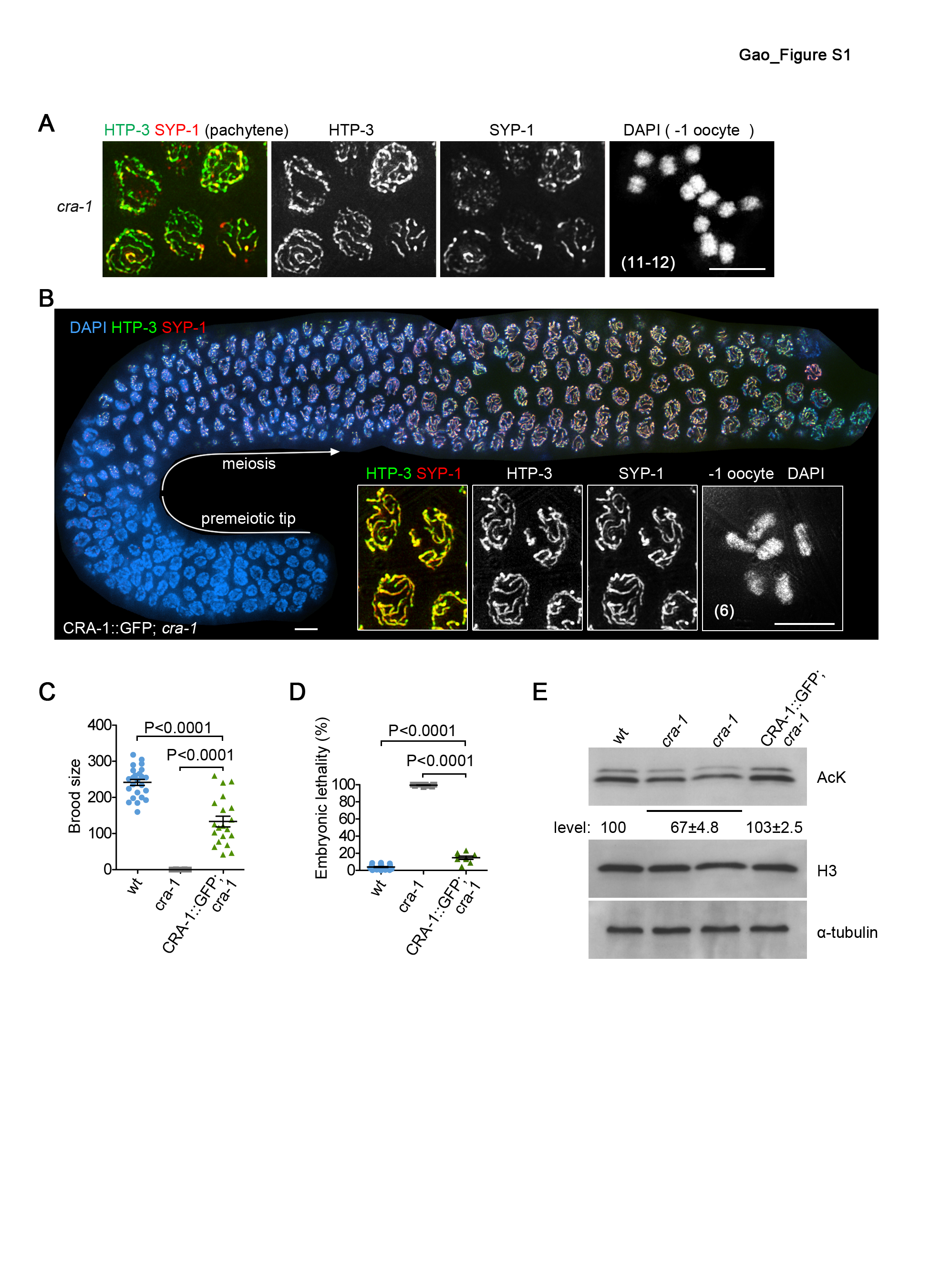

Supplement: S1 Fig — (A) Defects in synapsis and crossover formation in cra-1 mutants. Gonads from cra-1 mutant worms were immunostained for the axial and central region components of the SC, HTP-3 (green) and SYP-1 (red), respectively. DNA was stained with DAPI. Nuclei show discontinuous stretches of SYP-1 staining indicating unsynapsed chromosomes. 11 to 12 DAPI-stained bodies are observed in oocytes at diakinesis indicating lack of chiasmata. Bar, 5 μm. (B) CRA-1::GFP rescues the synapsis defects observed in cra-1 mutants. Gonads dissected from CRA-1::GFP; cra-1 worms were co-stained with anti-HTP-3 (green), anti-SYP-1 (red) and DAPI (blue). Insets: left and middle show pachytene nuclei with normal synapsis; right, six DAPI-stained bodies can be observed in the -1 oocyte corresponding to the six pairs of attached homologous chromosomes in C. elegans hermaphrodites. Bar, 5 μm. (C) Brood size is significantly increased in the CRA-1::GFP; cra-1 line compared to cra-1 mutants. * P<0.0001, two-tailed Mann-Whitney test, 95% C.I. (D) Embryonic lethality is reduced among the offspring of the CRA-1::GFP; cra-1 line compared to cra-1 mutants. * P<0.0001, two-tailed Mann-Whitney test, 95% C.I. (E) Global histone acetylation is rescued in the CRA-1::GFP; cra-1 line compared to cra-1 mutants. Anti-acetylated lysine antibody (AcK) was used to detect global histone acetylation. The levels of histone H3 and α-tubulin were used as loading controls. The relative level of acetylated histones was determined by densitometric analysis of the western blot bands (AcK vs. H3) using ImageJ. Numbers represent mean ± SEM for data from at least two independent experiments. (TIF) [file pgen.1005029.s001.tif]

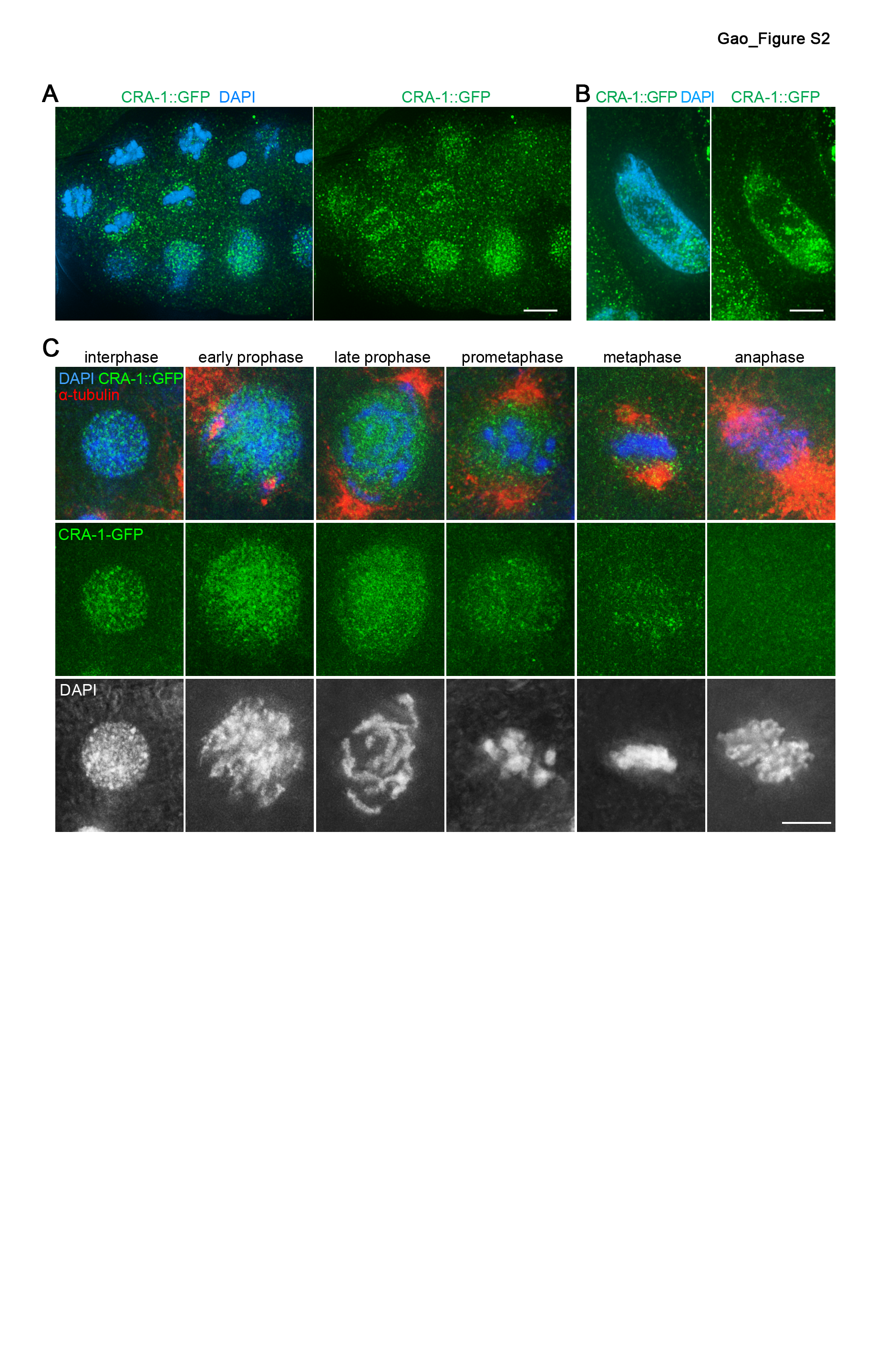

Supplement: S2 Fig — (A) Co-staining with an anti-GFP antibody (green) and DAPI (blue) in embryos from CRA-1::GFP transgenic adult worms. Bar, 5 μm. (B) Co-staining with an anti-GFP antibody (green) and DAPI (blue) of an intestinal nucleus from CRA-1::GFP transgenic adult worms. Bar, 5 μm. (C) CRA-1::GFP expression during embryonic cell cycle progression. CRA-1::GFP embryos were immunostained with anti-GFP antibody (green) and anti-α-tubulin antibody (red). DNA (blue) was stained with DAPI. Bar, 5 μm. (TIF) [file pgen.1005029.s002.tif]

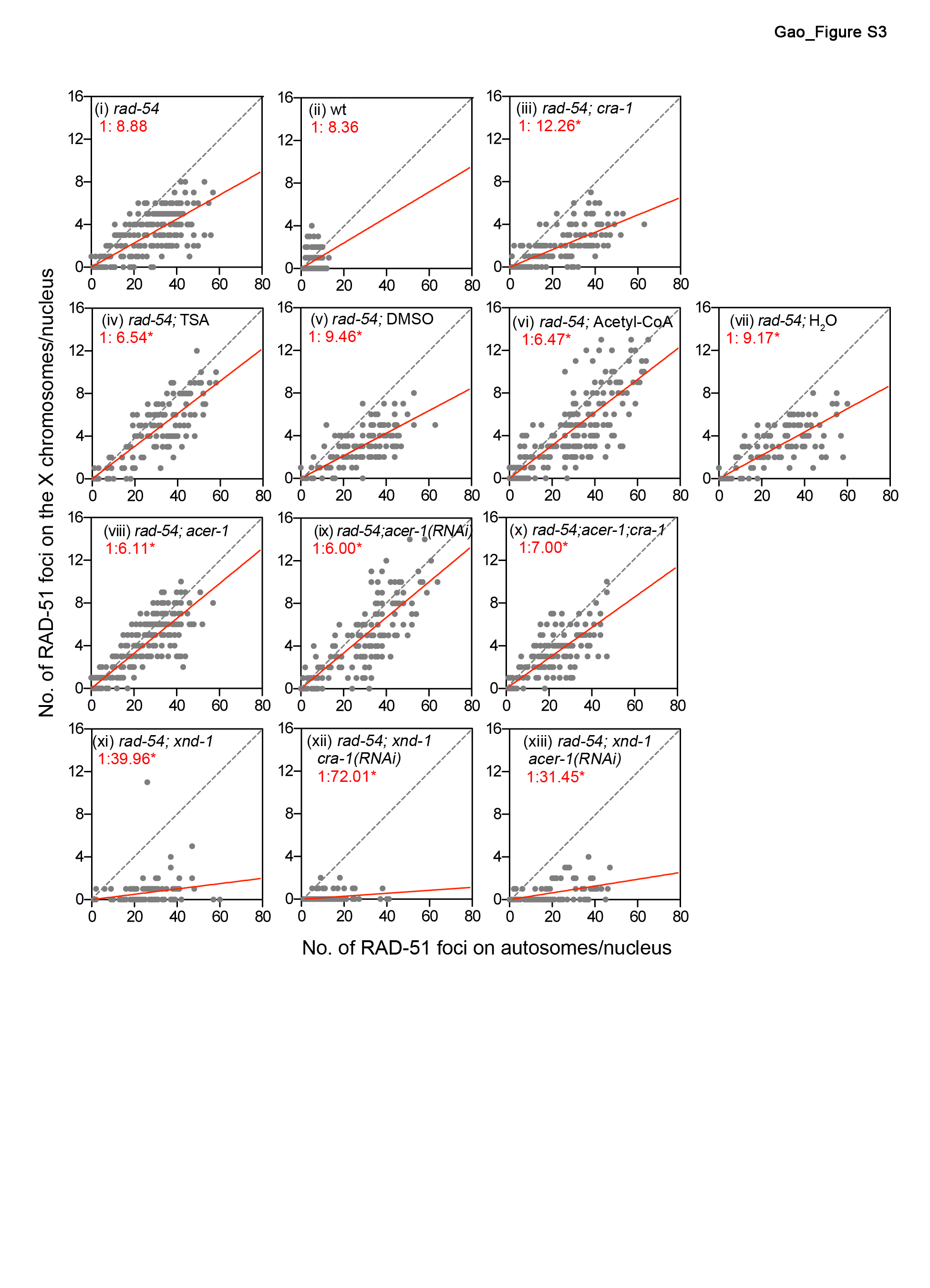

Supplement: S3 Fig — Graphs depict the distribution of RAD-51 foci levels detected on the X chromosomes and the autosomes during early meiotic prophase (from transition zone to mid pachytene all combined). The average ratios of DSBs inferred from the quantification of RAD-51 foci on the X versus autosomes are indicated. Dashed lines indicate a X/A ratio of 1:5. (TIF) [file pgen.1005029.s003.tif]

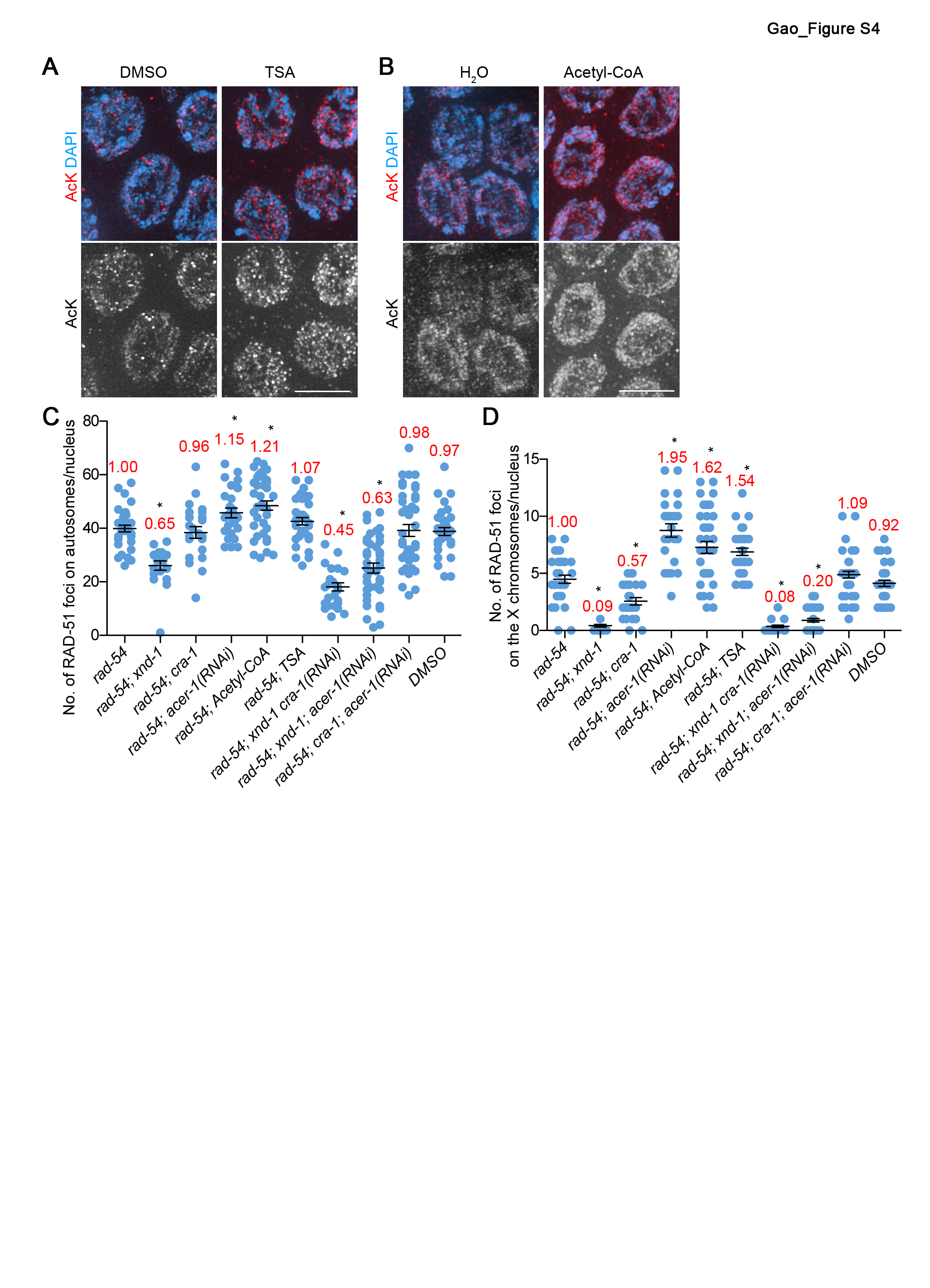

Supplement: S4 Fig — (A) Gonads from wild type worms injected with 10 μM TSA or 0.2% DMSO (v/v) were immunostained with a pan acetylation antibody (red) and DNA was stained with DAPI (blue). Shown are late pachytene nuclei. Bar, 5 μm. (B) Gonads from wild type worms injected with H2O or 100μM Acetyl-CoA were immunostained with a pan acetylation antibody (red) and DNA was stained with DAPI (blue). Shown are late pachytene nuclei. Bar, 5 μm. (C) Analysis of RAD-51 foci levels on autosomes in mid pachytene (zone 4) nuclei in the indicated genotypes. Bars represent the mean number ± SEM of RAD-51 foci observed on autosomes per nucleus. The fold changes in the mean numbers of RAD-51 foci on autosomes relative to rad-54 single mutants are indicated for each genotype (red numbers). * P≤0.0077, two-tailed Mann-Whitney test, 95% C.I. (D) Analysis of RAD-51 foci levels on the X chromosomes in mid pachytene (zone 4) nuclei in the indicated genotypes. Bars represent the mean number ± SEM of RAD-51 foci observed on the X chromosomes per nucleus. The fold changes in the mean numbers of RAD-51 foci on the X chromosomes relative to rad-54 single mutants are indicated for each genotype (red numbers). * P≤0.0155. (TIF) [file pgen.1005029.s004.tif]

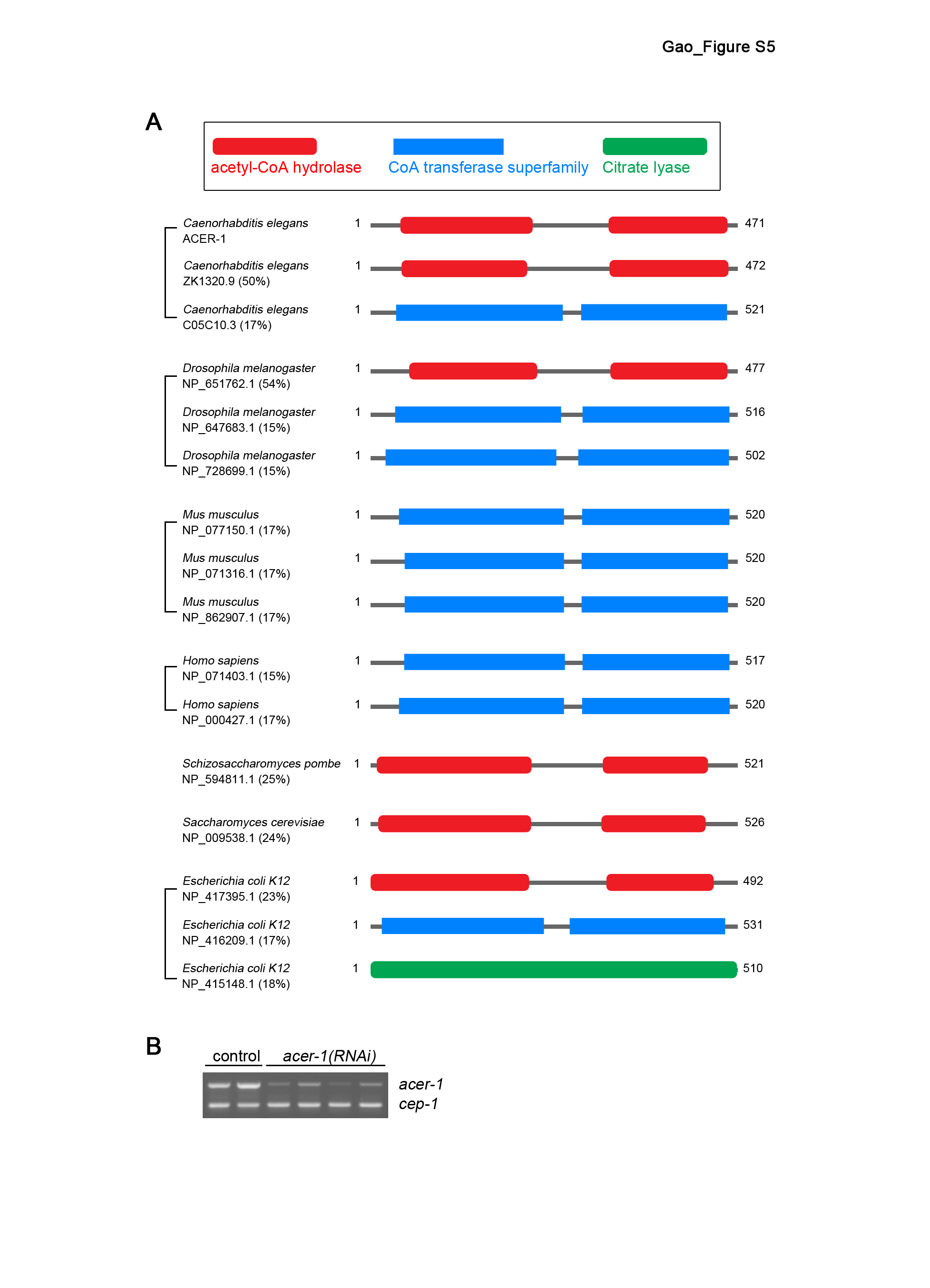

Supplement: S5 Fig — (A) ACER-1 homologs present from bacteria to humans. Homologs were identified through a HHPRED search, which is based on similarity both in sequences and structure. The acetyl-CoA hydrolase and CoA-transferase domains share a high degree of similarity in both sequence and structure, consistent with previous findings that an acetyl-CoA hydrolase domain may have both hydrolase and transferase activity [51,52]. (B) Single-worm RT-PCR analysis of acer-1(RNAi) worms. Depletion of ACER-1 was obtained by microinjection of acer-1 dsRNA. Single worm RT-PCR was performed to analyze RNAi efficiency comparing control (empty vector) worms to acer-1(RNAi) worms. cep-1/p53 was assessed as a loading control. (TIF) [file pgen.1005029.s005.tif]

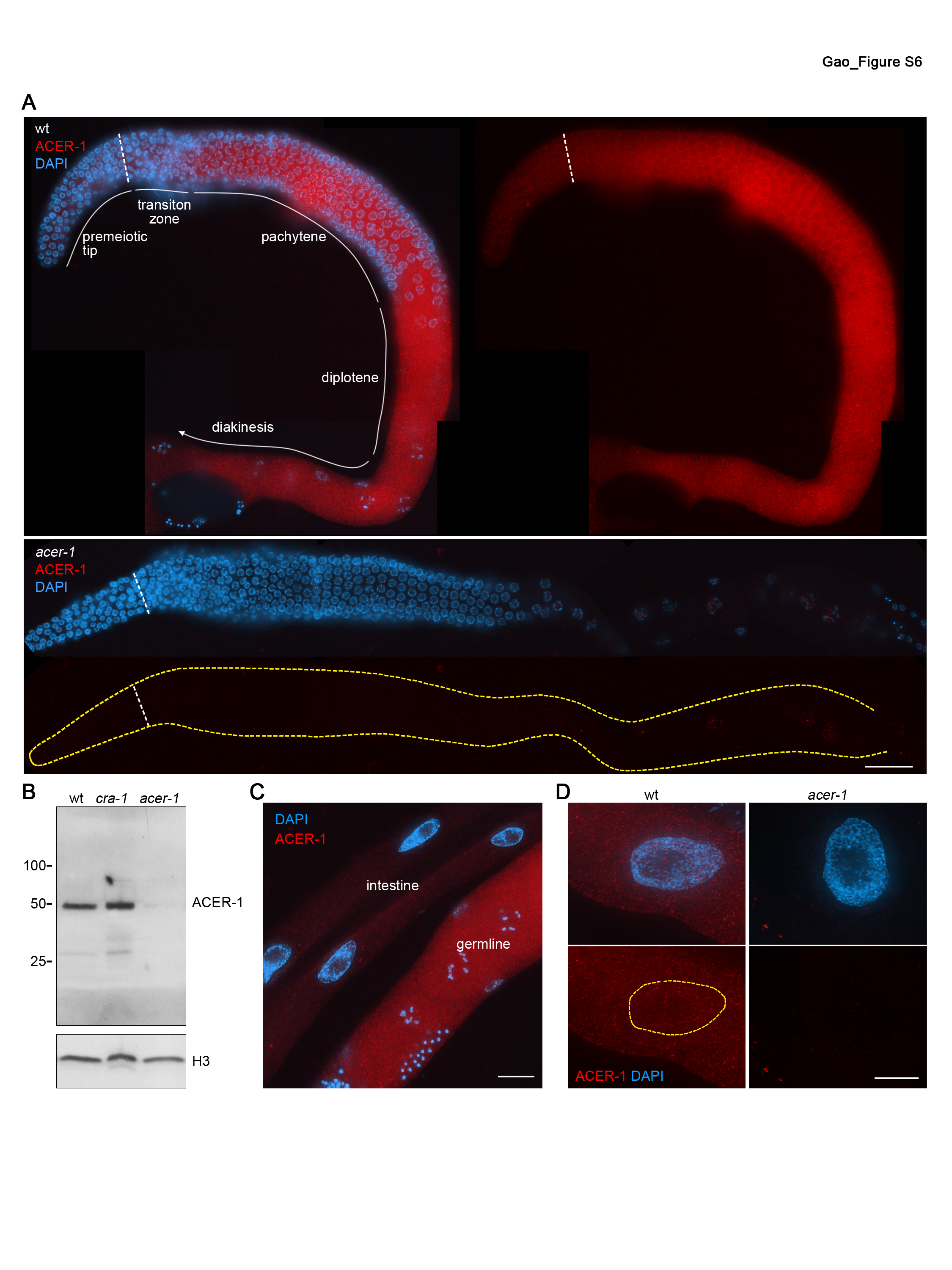

Supplement: S6 Fig — (A) Co-staining of wild type and acer-1 mutant germlines with an anti-ACER-1 antibody (red) and DAPI (blue). Gonads from wild type and acer-1 mutants were fixed and immunostained on the same slides. All images were captured under the same exposure conditions with the DeltaVision system (Applied Precision). Yellow dashed lines were utilized to facilitate visualization of the gonad’s outline when only the antibody signal is depicted. Bar, 30 μm. (B) Western blot analysis of ACER-1 and histone H3 in whole worm lysates from wild type, cra-1 and acer-1 mutants. (C) Image shows ACER-1 (red) immunostaining in wild type germline (diakinesis) and intestine. DNA was stained with DAPI (blue). Bar, 10 μm. (D) Co-staining of wild type and acer-1 mutant intestinal cells with an anti-ACER-1 antibody (red) and DAPI (blue). Bar, 5 μm. (TIF) [file pgen.1005029.s006.tif]

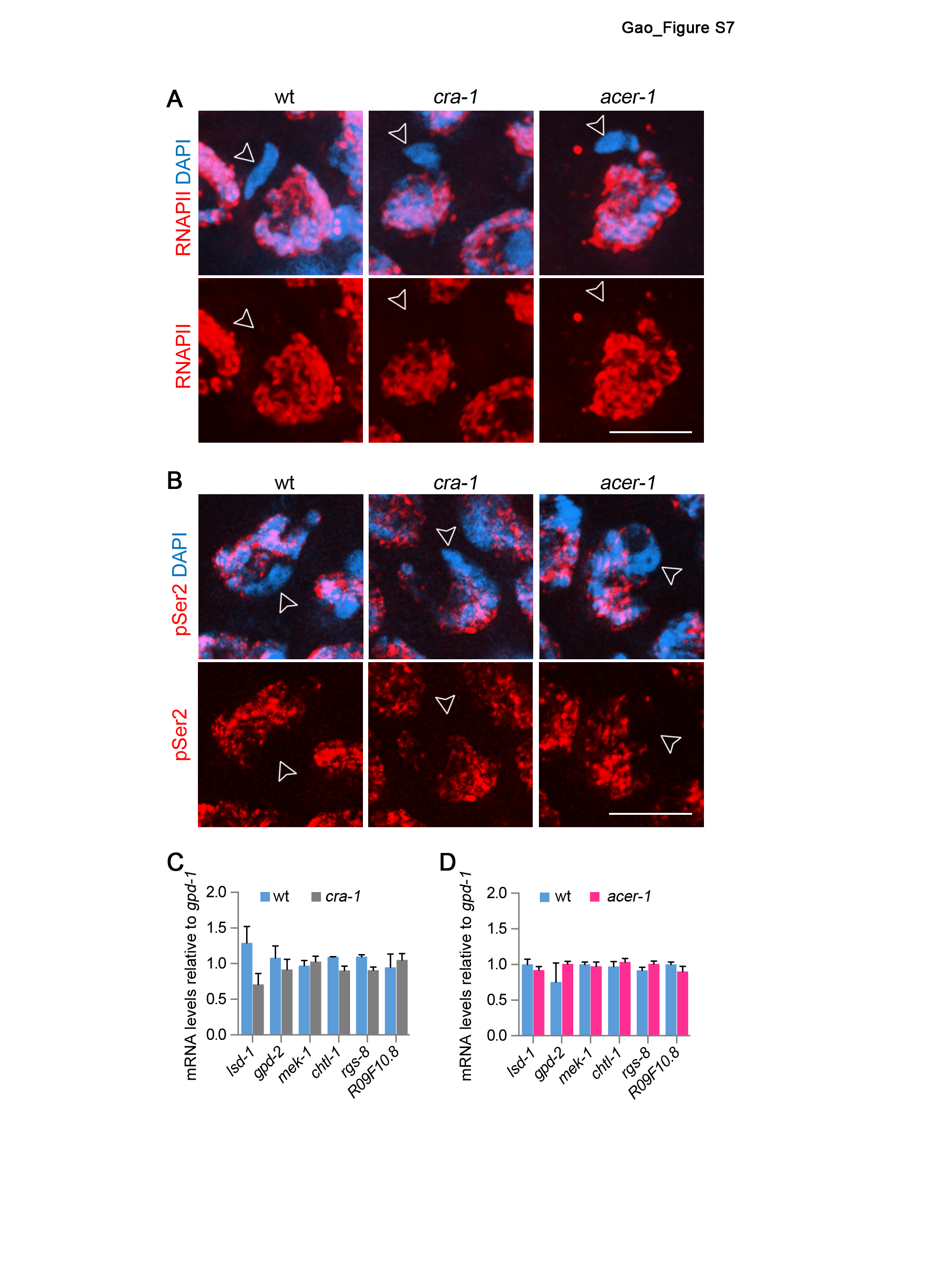

Supplement: S7 Fig — (A) Co-staining with anti-RNA polymerase II antibody (Covance, clone CTD4H8, 1:200) (red) and DAPI (blue) of early pachytene nuclei from the indicated genotypes. Open arrowheads indicate X chromosomes. The pattern of RNA Polymerase II localization between autosomes and the X chromosomes is not altered in cra-1 and acer-1 mutants compared to wild type. Bar, 3 μm. (B) Co-staining with pSer2 antibody (Covance, clone H5, 1:100) (red) and DAPI (blue) of early pachytene nuclei from the indicated genotypes. Open arrowheads indicate X chromosomes. The localization pattern of pSer2 between autosomes and the X chromosomes is not altered in cra-1 and acer-1 mutants compared to wild type. Bar, 3 μm. (C) Expression levels of six genes on the X chromosome from dissected gonads of wild type and cra-1 mutants. Quantitative RT-PCR analysis revealed that the germline-specific gene expression for the depicted genes was not altered when comparing cra-1 to wild type and normalizing to gpd-1. (D) Analysis was performed as in (C) except that acer-1 mutants were used instead of cra-1 mutants. Expression levels of the six genes were not altered in acer-1 mutants compared to wild type. (TIF) [file pgen.1005029.s007.tif]

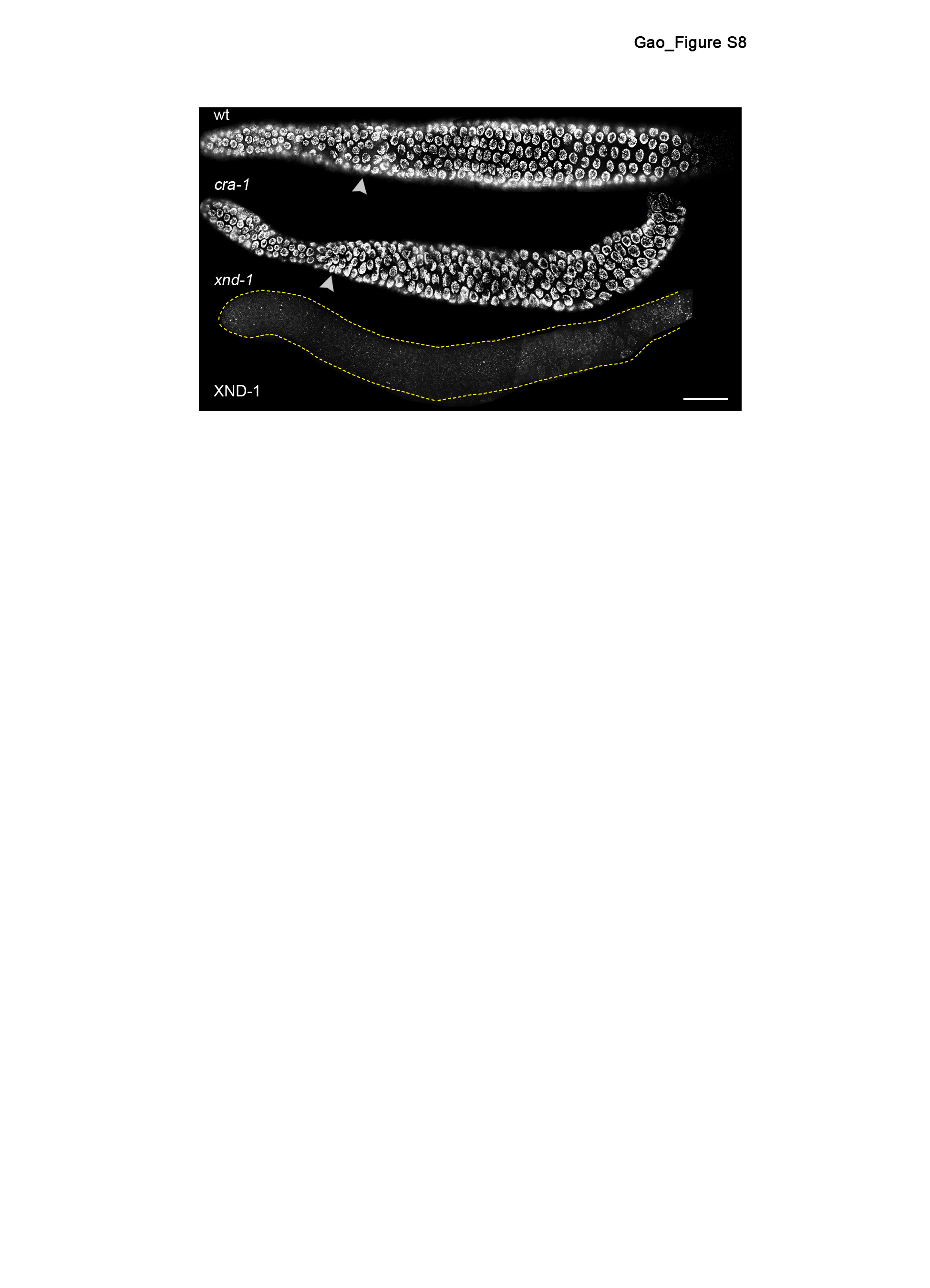

Supplement: S8 Fig — Gonads dissected from wild type, cra-1 and xnd-1 mutants were immunostained with an anti-XND-1 antibody. Gonads are oriented such that progression through meiosis is from left to right. White arrowheads indicate entrance into meiosis (beginning of the transition zone). Bar, 20 μm. (TIF) [file pgen.1005029.s008.tif]
