# Supplementary material for: NatB Domain-Containing CRA-1 Antagonizes Hydrolase ACER-1 Linking Acetyl-CoA Metabolism to the Initiation of Recombination during C. elegans Meiosis
Source: PLoS Genet. 2015 Mar 13;11(3):e1005029. doi: 10.1371/journal.pgen.1005029 (PMC4359108; doi:10.1371/journal.pgen.1005029)
Supplement: S1 Text — Immunoprecipitation from CRA-1::GFP whole worm lysates with an anti-GFP antibody was analyzed by mass spectrometry. Experiment was performed in triplicate. The potential CRA-1 interacting proteins that were identified in at least two of the experiments were listed. Numbers indicate the total mass spectra collected from 3 experiments. (DOCX) [file pgen.1005029.s009.docx]

**S1 Text**

**NatB domain-containing CRA-1 antagonizes hydrolase ACER-1 linking Acetyl-CoA metabolism to the initiation of recombination during *C. elegans* meiosis**

Jinmin Gao, Hyun-Min Kim, Andrew E. Elia, Stephen J. Elledge and Monica P. Colaiácovo*

Department of Genetics, Harvard Medical School, Boston, MA 02115

* Correspondence: [mcolaiacovo@genetics.med.harvard.edu](mailto:mcolaiacovo@genetics.med.harvard.edu); Phone: 617-432-6543; Fax: 617-432-7663.

**SUPPORTING PROTOCOL**

**Mass Spectrometry**

TCA-precipitated proteins were dissolved in 100mM ammonium bicarbonate (pH 8.0) with 10% acetonitrile and 10 ng/µL trypsin (Promega) and incubated at 37°C for 5 hours. They were subsequently desalted, dissolved in 5% formic acid / 5% acetonitrile, and loaded onto a reversed phase microcapillary column (100 mm I.D.) packed with 18 cm of Maccel C18AQ resin (3 mm, 200Å, The Nest Group, Inc). Peptides were eluted using a gradient of 4%–26% acetonitrile in 0.125% formic acid over 125 minutes and detected in a hybrid linear ion trap-orbitrap mass spectrometer (LTQ-Orbitrap Discovery, ThermoFisher). Precursors selected for MS/MS fragmentation were corrected for errors in monoisotopic peak assignment, and tandem MS spectra were searched using the Sequest algorithm, with mass tolerance set to 25 ppm and two missed cleavages allowed. False discovery rates were estimated with the target-decoy method [1], and linear discriminant analysis (LDA) was utilized to filter peptides to a 1% peptide-level FDR. Peptides were then assembled into proteins and further filtered to a protein-level FDR of 0.81% [2], resulting in a final peptide-level FDR of 0.12%.

**S1 Table. Potential CRA-1 interacting proteins**

| Total no. of peptides detected | Protein Name or ORF | Description |
| --- | --- | --- |
| 60 | rps-13 | 40S ribosomal protein S13 |
| 59 | hrpf-1 | Putative pre-mRNA splicing factor |
| **55** | **cra-1** | **Central Region Assembly in meiosis abnormal family member** |
| 35 | rps-6 | A small (40S) ribosomal subunit S6 protein |
| **33** | **acer-1** | **Potential Acetyl-CoA hydrolase/transferase** |
| 33 | rla-1 | 60S acidic ribosomal protein P1 |
| 31 | PRP-19 | Pre-mRNA-processing factor 19 homolog |
| 30 | rpl-9 | 60S ribosomal protein L9 |
| 26 | tlk-1 | Tousled-class serine/threonine protein kinase |
| 19 | W09D12.1 | Zinc metalloprotease |
| 12 | rack-1 | RACK1 (mammalian Receptor of Activated C Kinase) homolog |
| 11 | F40F4.6 | EGF-like domain |
| 11 | Y69H2.14 | Uncharacterized protein |
| 10 | Y75B8A.3 | Carboxylesterase, type B |
| 10 | dpy-18 | Prolyl 4-hydroxylase subunit alpha-1 |
| 10 | col-160 | Collagen |
| 8 | eef-2 | Translation elongation factor 2 |
| 8 | vbh-1 | Vasa-and belle-like helicase protein 1, isoform b |
| 8 | F23C8.6 | Uncharacterized protein |
| 7 | Y37E3.8 | Ribosomal protein L18e/L15P |
| 7 | col-119 | Collagen |
| 6 | SUCA-1 | Succinyl-CoA ligase |
| 6 | Y53G8AR.9 | ZF-CCCH super family protein |
| 6 | clic-1 | Clathrin light chain |
| 4 | imb-3 | Importin-beta-like protein |
| 4 | tag-174 | Probable cytochrome c oxidase subunit 6A |
| Immunoprecipitation from CRA-1::GFP whole worm lysates with an anti-GFP antibody was analyzed by mass spectrometry. Experiment was performed in triplicate. The potential CRA-1 interacting proteins that were identified in at least two of the experiments were listed. Numbers indicate the total mass spectra collected from 3 experiments. | | |

**Supporting References**

1. Elias JE, Gygi SP (2007) Target-decoy search strategy for increased confidence in large-scale protein identifications by mass spectrometry. Nat Methods 4: 207-214.

2. Huttlin EL, Jedrychowski MP, Elias JE, Goswami T, Rad R, et al. (2010) A tissue-specific atlas of mouse protein phosphorylation and expression. Cell 143: 1174-1189.
